# Determinants of metabolic syndrome in people living with human immunodeficiency virus in Africa: a systematic review and meta-analysis

**DOI:** 10.3389/frph.2025.1689731

**Published:** 2025-11-04

**Authors:** Emmanuel Agada David, Olatunde Ayodeji Olayanju, Kamaldeen Olalekan Sanusi, Oluseun Iyabode Mabadeje, Philemon Paul Mshelia, Ibuchukwu Orabueze, Chioma Nneka Kunle-Ope, Ifeyinwa Ezenwosu, Kasimu Mamuda, Sonnen Atinge, Adeoti Gbemisola Adeniran, Saheed Olatunbosun Akiode, Oluchukwu Perpetual Okeke, Olunike Rebecca Abodunrin, Folahanmi Tomiwa Akinsolu, Olajide Odunayo Sobande

**Affiliations:** ^1^ Department of Clinical Pharmacy and Pharmacy Practice, Faculty of Pharmaceutical Sciences, Gombe State University, Tudun Wada, Nigeria; ^2^ Department of Chemical Pathology, Ben Carson College of Health and Medical Sciences, Babcock University, Ilisan-remo, Nigeria; ^3^ Department of Human Physiology, Faculty of Basic Medical Sciences, College of Health Sciences, Al-Hikmah University, Ilorin, Nigeria; ^4^ Department of Microbiology, Southwestern University, Okun-owa, Nigeria; ^5^ Department of Human Physiology, College of Medical Sciences, Abubakar Tafawa Balewa University, Bauchi, Nigeria; ^6^ Department of Medical Microbiology, College of Medicine, University of Nigeria Ituku-Ozalla Campus, Enugu, Nigeria; ^7^ Department of Microbiology, Centre for Tuberculosis Research, Nigeria Institute of Medical Research, Lagos, Nigeria; ^8^ Department of Community Medicine, University of Nigeria Teaching Hospital, Ituku-Ozalla Campus, Enugu, Nigeria; ^9^ National Tuberculosis and Leprosy Training Centre, Zaria, Nigeria; ^10^ Department of Community Medicine, Federal University, Wukari, Nigeria; ^11^ Department of Physiology, University of Medical Sciences, Ondo, Nigeria; ^12^ Biotechnology Advanced Research Centre, Sheda Science and Technology Complex (SHESTCO), Abuja, Nigeria; ^13^ Nigerian Institute of Medical Research Foundation, Yaba, Nigeria; ^14^ Department of Epidemiology and Biostatics, Nanjing Medical University, Nanjing, China; ^15^ Department of Research, Capacity Building and Implementation, Alabastron Initiative, Lagos, Nigeria; ^16^ Center for Reproduction and Population Studies, Clinical Sciences Department, Nigerian Institute of Medical Research, Yaba, Nigeria; ^17^ Department of Public Health, Faculty of Basic Medical and Health Sciences, Lead City University, Ibadan, Nigeria

**Keywords:** metabolic syndrome, Africa, risk factors, people living with human immunodeficiency virus, sex, alcohol consumption, body mass index

## Abstract

**Background:**

Metabolic syndrome (MetS) among people living with HIV (PLHIV) is an emerging concern in Africa, but its underlying causes remain unclear. This study is a systematic review and meta-analysis of observational studies published between January 2000 and June 2025 to synthesize evidence on the determinants of MetS among PLHIV in Africa.

**Methods:**

PubMed, Web of Science, Scopus, and Cumulative Index to Nursing and Allied Health Literature (CINAHL) databases were searched for studies reporting determinants of MetS among PLHIV in Africa. Two reviewers independently screened and extracted data, and the risk of bias was assessed with the Newcastle-Ottawa Scale.

**Results:**

Thirty-six studies were included, while 23 were meta-analyzed. Female sex was strongly associated with MetS [Pooled odds ratios (PORs) = 2.86, 95% CI: 1.74–4.72], as was alcohol consumption (POR = 1.46, 95% CI: 1.04–2.03) and elevated BMI (>25 kg/m^2^) (POR = 4.27, 95% CI: 1.83–9.33). HIV-positive status showed significant effect (OR = 1.04, 95% CI: 1.01–1.09), while smoking (POR = 0.88, 95% CI: 0.48–2.70) and physical activity (POR = 0.98, 95% CI: 0.35–2.80) were not significantly associated. Substantial heterogeneity was observed for BMI, smoking, and physical activity.

**Conclusion:**

Female sex, alcohol consumption, and elevated BMI emerged as consistent determinants of MetS among PLHIV in Africa. These findings highlight the importance of proactively integrating, context-specific strategies for metabolic risk management into HIV care to address the rising burden of cardiometabolic disease in the region.

**Systematic Review Registration:**

https://www.crd.york.ac.uk/PROSPERO/view/CRD420251066865, PROSPERO CRD420251066865.

## 1 Introduction

Over the past few decades, the public health response to HIV/AIDS in Africa has undergone a profound transformation (1). Since the global peak in 1996, HIV incidence has declined by nearly 50% (2, 3). The scale-up of antiretroviral therapy (ART) has converted HIV from a fatal disease into a manageable chronic condition (4). With expanded ART coverage, millions of people living with HIV (PLHIV) in Africa now experience improved survival and quality of life (5). By 2024, over 31 million people worldwide were on ART, substantially reducing AIDS-related mortality (3). While this longevity is a major public health achievement, it has introduced new health challenges (6, 7). Among the most critical of these is the rising burden of non-communicable diseases (NCDs), including metabolic syndrome (MetS), which now contributes significantly to morbidity and mortality among PLHIV (8).

MetS is a cluster of interrelated cardiometabolic abnormalities, abdominal obesity, insulin resistance or glucose intolerance, elevated triglycerides, low high-density lipoprotein (HDL) cholesterol, and hypertension, that increase the risk of type 2 diabetes, cardiovascular disease (CVD), and stroke (9). Its presence is linked to a two- to three-fold increase in cardiovascular morbidity and mortality (10, 11). Among PLHIV, the pathogenesis of MetS is multifactorial. HIV infection itself induces chronic inflammation and immune dysregulation, even in individuals with viral suppression, thereby accelerating metabolic dysfunction (6, 12). Additionally, long-term exposure to certain ART regimens, especially protease inhibitors and thymidine analogues has been associated with insulin resistance, dyslipidemia, and lipodystrophy (13). These treatment-related effects may act synergistically with other risk factors common in African settings, such as poor diet, sedentary lifestyle, tobacco use, and viral co-infections like hepatitis B or C (14, 15).

In Africa, the dynamics of HIV and MetS are shaped by distinct epidemiological and structural factors. Rapid demographic and nutritional transitions, driven by urbanization, changing diets, and reduced physical activity, are fueling obesity and NCD risk (16, 17). Genetic predispositions may also influence vulnerability, though evidence is still emerging (18). Meanwhile, health systems remain largely designed for infectious disease control, with limited integration of NCD care (19, 20). Resource constraints, diagnostic gaps, and workforce shortages further hinder effective screening and management. ART regimen availability also differs from that of high-income countries; while integrase inhibitors like dolutegravir are increasingly adopted, many patients continue on older regimens associated with higher metabolic risks (21). These contextual factors underscore the importance of region-specific evidence to inform prevention and care strategies.

Despite growing recognition of these challenges, evidence on the determinants of MetS among African PLHIV remains fragmented. Studies differ widely in design, populations, diagnostic criteria (e.g., NCEP ATP III, IDF, and WHO), and the risk factors assessed, making cross-comparisons difficult (22, 23). While individual studies have identified potential determinants such as age, sex, body mass index (BMI), ART duration, alcohol use, physical inactivity, and socio-economic factors, their relative importance and consistency are unclear. Without a consolidated understanding, prevention and intervention strategies may be ineffective or misdirected.

Previous reviews of MetS in PLHIV often fail to account for Africa's unique epidemiological, therapeutic, and socio-economic contexts. To our knowledge, no synthesis has systematically quantified the determinants of MetS among African PLHIV while accounting for regional lifestyle transitions and ART patterns. This review addresses that gap by pooling evidence across the continent and applying meta-analytic methods to identify consistent determinants. The findings aim to provide actionable, context-specific insights for policymakers, clinicians, and researchers to better integrate NCD prevention within HIV care across Africa.

## 2 Methods

### 2.1 Study protocol

This systematic review and meta-analysis were carried out according to a pre-registered protocol on PROSPERO (CRD420251066865). The review process adhered to the Preferred Reporting Items for Systematic Reviews and Meta-Analyses (PRISMA) 2020 guidelines and the PRISMA extension for meta-analyses of observational studies (24) (Supplementary Table S1). To ensure methodological rigor, all stages of the review, including study selection, data extraction, and quality assessment, were performed independently by two reviewers. All disagreements were resolved through discussion, and where consensus was not achieved, a third reviewer acted as arbitrator.

### 2.2 Search strategy

A comprehensive literature search was conducted across four electronic databases: PubMed, Scopus, Web of Science, and CINAHL. To ensure completeness, we also performed hand searches for reference lists from all eligible and relevant articles. The search covered studies published between January 2000 and June 2025.

The strategy combined both controlled vocabulary (e.g., MeSH terms) and free-text keywords related to metabolic syndrome and its synonyms (e.g., “*metabolic syndrome,” “dysmetabolic syndrome,” “cardiometabolic syndrome,” “syndrome X,”* and “*insulin resistance syndrome”*) in conjunction with terms for HIV and its synonyms. Searches were restricted to studies conducted in African settings.

The detailed search strategies for each database are provided in the (Supplementary Table S2). No language restrictions were applied.

### 2.3 PICOS framework

The eligibility criteria for this review were structured using the PICOS (Population, Intervention/Exposure, Comparator, Outcomes, Study design) framework (Table 1). This approach provided a transparent and systematic way of defining study inclusion and exclusion parameters.

**Table 1:** PICOS framework.

Element	Description
Population	PLHIV of any age residing in African countries.
Intervention/exposure	Determinants or risk factors for metabolic syndrome, including sociodemographic (e.g., age, sex), behavioral (e.g., alcohol use, smoking, physical activity), clinical (e.g., BMI, hypertension, lipid abnormalities), and treatment-related factors (e.g., ART type or duration).
Comparator	HIV-negative controls, or within-population comparisons such as exposed vs. unexposed groups (e.g., alcohol users vs. non-users, overweight vs. normal BMI). Studies without explicit comparator groups were also eligible if effect estimates were provided.
Outcomes	Presence or absence of metabolic syndrome as defined by recognized diagnostic criteria (NCEP ATP III, IDF, WHO, or JIS).
Study design	Observational designs, specifically cross-sectional, case–control, and cohort studies. Excluded were qualitative studies, case reports, case series, reviews, editorials, and opinion pieces.

### 2.4 Eligibility criteria

Studies were considered eligible if they:

1. Included PLHIV of any age residing in African countries.
2. Examined determinants or risk factors associated with MetS.
3. Employed an observational study design, specifically cross-sectional, case–control, or cohort studies.
4. Were published between January 2000 and June 2025.

We excluded studies conducted outside Africa, those not involving PLHIV as the study population, articles that did not report determinants of MetS, as well as qualitative studies, case reports, case series, editorials, and opinion papers.

### 2.5 Study selection

**Figure 1:**
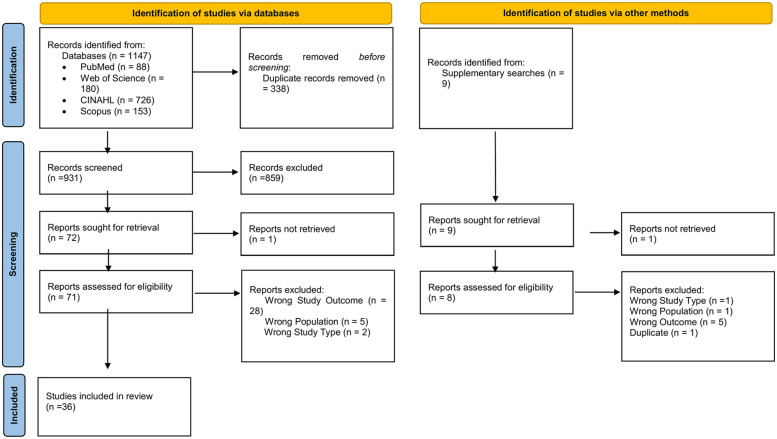
PRISMA flow diagram.

All records retrieved from the database searches were imported into Rayyan, a web-based tool designed for systematic review management, where duplicates were automatically removed (25). The selection process was conducted in two stages. In the first stage, two reviewers (OAO and EAD) independently screened titles and abstracts against the eligibility criteria to identify potentially relevant studies. Articles that did not meet the inclusion criteria (e.g., wrong population, non-African setting, or non-observational design) were excluded at this stage. In the second stage, full texts of all potentially eligible articles were obtained and reviewed independently by the same two reviewers (OAO and EAD). Each article was assessed for population, study design, outcome measures, and reporting of MetS determinants. Disagreements at either stage were resolved through discussions. When consensus could not be reached, a third reviewer acted as an adjudicator (KOS). Reasons for exclusion or inclusion at the full-text stage were systematically documented and are presented in (Supplementary Table S3). The overall selection process is summarized in the PRISMA flow diagram (Figure 1).

### 2.6 Data extraction

Data extraction was conducted independently by two reviewers (PPM and OM) using a standardized and piloted form developed in Google sheet, following Cochrane guidance (26). To ensure accuracy and completeness, the extraction form was first pilot tested on a small sample of eligible studies and refined before full data collection. For each study, we collected information on key identifiers, including the first author, year of publication, country, and geographical region, as well as details of the study design, setting, and total sample size. Participant-level characteristics such as mean or median age, sex distribution, and ART status were also recorded.

Information on the diagnosis of MetS was carefully extracted, including the criteria used (e.g., NCEP ATP III, IDF, WHO, or JIS) and how these definitions were operationalized within the study. Determinants of MetS were grouped into sociodemographic, behavioral, and clinical categories, and variables such as alcohol consumption, smoking, physical activity, BMI, lipid profile, and blood pressure were noted. For each determinant, effect size estimates (odds ratios, relative risks, or hazard ratios) with corresponding 95% confidence intervals were extracted. When studies reported multiple models, preference was given to multivariable-adjusted estimates, while unadjusted values were only included if no adjusted estimates were available.

To enhance transparency, additional information such as funding sources, study limitations noted by authors, and any declared conflicts of interest were also documented. Both reviewers cross-checked the extracted data for consistency. Any discrepancies were resolved through discussion, and in cases where consensus was not achieved, a third reviewer (KOS) served as an adjudicator. Where relevant, attempts were made to contact study authors for clarification or to obtain missing data. Details of the extraction are presented in (Supplementary Table S3).

### 2.7 Quality and risk of bias assessment

The methodological quality and risk of bias of the included studies were assessed using the star-based Newcastle-Ottawa Scale (NOS), adapted for cross-sectional studies (27). Each study was evaluated across three domains: selection, comparability, and outcome assessment. The selection domain carried a maximum of four stars, which were awarded for representativeness of the exposed cohort, adequacy of sample size justification, ascertainment of exposure, and reporting of non-respondents or missing data. The comparability domain had a maximum of two stars and was used to evaluate whether studies controlled for important confounding variables; two stars were allocated for adequate control, while only one was awarded in cases of partial or limited adjustment. The outcome domain allowed a maximum of three stars, assessing whether outcomes were measured reliably, whether appropriate statistical tools were applied, and whether outcome assessments were reported (Supplementary Tables S4).

### 2.8 Data synthesis and analysis

We first conducted a descriptive synthesis of the included studies, summarizing their geographical distribution, methodological approaches, diagnostic criteria for MetS, and the frequency of occurrence of the determinants. A meta-analysis was then performed when studies were deemed sufficiently homogeneous in terms of design, outcome definitions, and statistical reporting. All statistical analyses were conducted in R version 4.4.1 (R Foundation for Statistical Computing) using the *meta* and *metafor* packages. For each determinant, we extracted effect estimates [odds ratios, (ORs)] and synthesized them using a random-effects model (DerSimonian–Laird method) (28). The random-effects approach was chosen *a priori* because we anticipated substantial differences between-study heterogeneity, given the diversity of African study populations, variations in ART regimens, and differences in diagnostic criteria for MetS (29). Unlike a fixed-effects model, which assumes a single true effect size, the random-effects model accounts for variability across studies and provides more conservative pooled estimates, making it more appropriate for our dataset.

Effect estimates were presented as pooled odds ratios (PORs) with 95% confidence intervals (CIs). Determinants were only meta-analyzed when at least three studies reported on the same factor at a comparable level of measurement, ensuring consistency and reliability. Studies reporting hazard ratios (HRs) were excluded from pooled analyses unless three or more studies reported them for the same determinant, as combining disparate effect measures (HRs, ORs, RRs) can bias results.

Statistical heterogeneity was assessed using both Cochran's *Q* test, which tests the null hypothesis of homogeneity, and the I^2^ statistic, which quantifies the percentage of variability attributable to between-study differences rather than chance (30). We classified heterogeneity using standard thresholds (25% = low, 50% = moderate, 75% = substantial). Reporting both Q and I^2^ allowed us to capture both statistical significance and magnitude of heterogeneity, which is important given the diversity of African cohorts.

To assess publication bias, we constructed funnel plots for each determinant and visually inspected them for asymmetry. In addition, we applied the Trim and Fill method to adjust pooled estimates where asymmetry suggested possible bias from unpublished studies. This approach provides a more robust assessment of potential selective reporting (31).

Sensitivity analyses were conducted using a leave-one-out method, in which each study was sequentially removed to evaluate its influence on the overall pooled estimate (32). This approach allowed us to test the robustness of our findings and identify any single studies that disproportionately influenced results.

Statistical significance was defined as *p* < 0.05 for all analyses.

### 2.9 GRADE assessment

The certainty of evidence for each determinant of MetS was assessed using the Grading of Recommendations Assessment, Development, and Evaluation (GRADE) approach (33, 34). Under the GRADE framework, observational studies were initially rated as *low certainty* evidence but could be upgraded or downgraded depending on predefined criteria. Evidence was downgraded for serious risk of bias, inconsistency (heterogeneity across studies, *I*^2^ > 50%), indirectness (e.g., surrogate measures of determinants), imprecision (wide 95% CIs or small sample sizes), and potential publication bias (Egger's test and funnel plot asymmetry). Conversely, evidence was upgraded when studies demonstrated large effect sizes (OR ≥ 2.0 or ≤ 0.5), dose–response relationships, or when all plausible residual confounding would reduce, rather than increase, the observed effect.

The final certainty of evidence for each determinant was graded as high, moderate, low, or very low. A “Summary of Findings” (SoF) table was constructed following Cochrane and RevMan style to transparently present effect estimates, participant numbers, and certainty ratings (35). Details of GRADE assessment are presented in (Supplementary Table S7).

## 3 Results

### 3.1 Study selection

The initial database search identified a total of 1,147 records: 88 from PubMed, 180 from Web of Science, 726 from CINAHL, and 153 from Scopus. After removing 338 duplicates, 931 unique records were retained for screening. Title and abstract screening led to the exclusion of 859 articles, primarily due to ineligible study type, population, or design. Seventy-two articles were retrieved for full-text assessment. Of these, 36 were excluded, with 28 reporting outcomes unrelated to MetS, 5 involved populations outside the eligibility criteria, 2 used ineligible study designs, and 1 had full text which could not be accessed. Ultimately, 36 studies met the inclusion criteria and were retained for this review. The selection process is summarized in the PRISMA flow diagram (Figure 1).

### 3.2 Study characteristics

A total of 36 studies (36–71) conducted across 14 African countries were included in this review, with one study spanning both Zambia and Zimbabwe (43) (Table 2). Most of the studies employed a cross-sectional design (27/36; 75.0%) (36–41, 43, 44, 47–50, 52–65, 67), while five were cohort studies (5/36; 13.9%) (46, 66, 68–70) and four were case–control studies (4/36; 11.1%) (42, 45, 51, 71).

**Table 2:** Characteristics of included studies.

S/N	Author/ Publication Year	Country	Region	Study setting	Study design	Sex of participants	Age mean or range	Sample size	HIV +	HIV −	HIV& MetS	Prevalence (%)	MetS criteria
1	Abdela et al., 2023 (36)	Ethiopia	East Africa	2 Health facilities	Cross-sectional	Male and female	Mean (SD) yrs 45 (11)	518	518	Nil	NCEP/ATP III (195), IDF (193)	NCEP/ATP III (38), IDF (37)	NCEP/ATP III and IDF
2	Amutuhaire, 2023 (37)	Uganda	East Africa	1 Health facility	Cross-sectional	Male and female	Median (IQR) 31 (27–39)	309	309	Nil	43	14	IDF
3	Aouam et al., 2021 (38)	Tunisia	North Africa	1 Health facility	Cross-sectional	Male and female	Mean: Particiants with MetS (43.7), Without MetS (36.7)	70	70	Nil	19	27	IDF
4	Awotedu et al., 2010 (39)	South Africa	Southern Africa	1 Health facility	Cross-sectional	Male and female	Mean (SD) yrs ART-HIV+: 37.7 (9.2) Non-ART-HIV+: 36.0 (11.6) HIV-: 36.3 (13.7)	321	196	125	NCEP/ATP III (147) and IDF (157)	NCEP/ATP III (75) and IDF (80)	NCEP/ATP III and IDF
5	Berhane et al., 2012 (40)	Ethiopia	East Africa	1 Health facility	Cross-sectional	Male and female	≥18	313	313	Nil	66	21	NCEP/ATP III
6	Bosho et al., 2018 (41)	Ethiopia	East Africa	1 Health facility	Cross-sectional	Male and female	Mean (39.32) years	268	268	Nil	NCEP-ATP III (63), IDF (55), JIS (74)	NCEP-ATP III (24), IDF (21), JIS (28)	NCEP/ATP III, IDF, & JIF
7	Bune et al., 2020 (42)	Ethiopia	East Africa	4 Health facilities	Case-control	Male and female	Range: 18–70	633	633	Nil	139	22	NCEP/ATP III
8	Chihota et al., 2022 (43)	Zambia and Zimbabwe	Southern Africa	3 Health facilities	Cross-sectional	Male and female	Median (IQR) years HIV+: 40 (34–45) HIV−: 38 (32–46)	901	420	481	76	18	IDF
9	Dirajlal-Fargo et al., 2025 (44)	South Africa	Southern Africa	Cape Town Adolescents Antiretroviral Cohort	Cross-sectional	Male and female	Median (IQR) 18 (17–20)	364	293	71	48	16	IDF
10	Dzudzor et al., 2023 (45)	Ghana	West Africa	1 Health facility	Case-control	Male and female	Mean (38.4) years	464	308	156	102	33	JIS
11	Eholi et al., 2015 (46)	Côte d'Ivoire	West Africa	1 Health facility	Cohort Study	Male and female	Median (IQR) 35.7 (9.7)	176	176	Nil	31	18	IDF
12	Gebrie, 2020 (47)	Ethiopia	East Africa	2 Health facilities	Cross-sectional	Male and female	Mean (SD) 38.6 (10.3) years	407	407	Nil	100	25	IDF
13	Guira et al., 2016 (48)	Burkina-Faso	West Africa	1 Health facility	Cross-sectional	Male and female	Mean (SD) years 44.8 (7.4)	300	300	Nil	54	18	IDF
14	Hamoova et al., 2021 (49)	Zambia	Southern Africa	24 Health facilities	Cross-sectional	Male and female	Median (IQR) years 41 (34–49)	1,108	1,108	Nil	291	26	JIS
15	Hirigo and Tesfaye, 2016 (50)	Ethiopia	East Africa	1 Health facility	Cross-sectional	Male and female	Median (IQR) 35 (28.2–40.0)	185	185	Nil	NCEP/ATP III (33) and IDF (45)	NCEP/ATP III (18) and IDF (24)	NCEP/ATP III and IDF
16	Jarso et al., 2024 (51)	Ethiopia	East Africa	1 Health facility	Case-control	Male and female	Range: 40–60 years	393	393	Nil	131	33	JIS
17	Jemal et al., 2024 (52)	Ethiopia	East Africa	1 Health facility	Cross-sectional	Male and female	≥18	172	172	Nil	32	19	IDF
18	Jumare et al., 2023 (53)	Nigeria	West Africa	1 Health facility	Cross-sectional	Male and female	Median HIV+: 45 yrs HIV−: 40 yrs	672	440	232	135	31	NCEP/ATP III
19	Katoto et al., 2018 (54)	Democratic Republic of Congo	Central Africa	3 Health facilities	Cross-sectional	Male and female	Median (IQR) 43 (36–51)	495	495	Nil	134	27	NCEP/ATP III
20	Kiama et al., 2018 (55)	Kenya	East Africa	1 Health facility	Cross-sectional	Male and female	≥18	360	360	Nil	69	19	JIS
21	Labhardt et al., 2017 (56)	Lesotho	Southern Africa	10 Health facilities	Cross-sectional	Male and female	Median (IQR) 44.4 (35.3; 54.4)	1,166	1,166	Nil	195	17	IDF
22	Longo-Mbenza et al., 2015 (57)	Democratic Republic of Congo	Central Africa	1 Health facility	Cross-sectional	Male and female	Mean (SD) 42 (9) years.	116	116	Nil	61	53	IDF
23	Malindisa et al., 2023 (58)	Tanzania	East Africa	1 Health facility	Cross-sectional	Male and female	44 (Mean) years	223	223	Nil	51	23	IDF
24	Masyoko et al., 2020 (59)	Kenya	East Africa	1 Health facility	Cross-sectional	Male and female	Median(IQR) HIV+: 45 (39.5–53.0) HIV−: 40 (31–55)	598	300	298	19	6	Consensus Criteria 2009
25	Mirai et al., 2024 (60)	Tanzania	East Africa	1 Health facility	Cross-sectional	Male and female	Median (IQR) 54 (54–61) years	312	312	Nil	132	42	IDF
26	Moller et al., 2020 (61)	Ethiopia	East Africa	3 Health facilities	Cross-sectional	Male and female	Mean (33) Range (18–69) yrs	429	329	100	59	18	JIS
27	Muhammad et al., 2017 (62)	Nigeria	West Africa	1 Health facility	Cross-sectional	Male and female	Mean (34.8) years	300	300	Nil	29	10	NCEP/ATP III
28	Ngugen et al., 2021 (63)	South Africa	Southern Africa	17 Health facilities	Cross-sectional	Male and female	Mean (SD) 38.6 (9.0)	709	709	Nil	200	28	JIS
29	Ojong et al., 2022 (64)	Nigeria	West Africa	1 Health facility	Cross-sectional	Male and female	Mean: 39.2 years	225	150	75	NCEP/ATP III (29), IDF (58)	NCEP/ATP III (13), IDF (26)	NCEP/ATP III and IDF
30	Osoti et al., 2018 (65)	Kenya	East Africa	1 Health facility	Cross-sectional	Male and female	Median (IQR) years 40 (33–46)	300	300	Nil	48	16	IDF
31	Sobieszczyk et al., 2016 (66)	South Africa	Southern Africa	CAPRISA002 Acute HIV infection cohort	Cohort	Female	Median (IQR) 24 (21–28)	160	160	Nil	42	26	NCEP/ATP III
32	Tesfaye et al., 2014 (67)	Ethiopia	East Africa	1 Health facility	Cross-sectional	Male and female	Mean (SD) years ART-HIV+: 32.7 (9.7) Non-ART-HIV+: 32.6 (7.8)	374	374	Nil	NCEP/ATP III (63) and IDF (89)	NCEP/ATP III (17) and IDF (24)	NCEP/ATP III and IDF
33	Torpey et al., 2025 (68)	Ghana	West Africa	5 Health facilities	Cohort study	Male and female	Mean (SD) 45.31 (11.84)	2,512	2,512	Nil	961	38	JIS
34	Woldu et al., 2022 (70)	Ethiopia	East Africa	1 Health facility	Cohort study	Male and female	Mean (SD) yrs HIV+: 43.51 (11.27) HIV-: 50.74 (14.31)	510	288	222	NCEP/ATP III (82), IDF (126)	NCEP/ATP III (28), IDF (44)	NCEP/ATP III and IDF
35	Woldu et al., 2022 (69)	Ethiopia	East Africa	1 Health facility	Cohort study	Male and female	Mean (SD) yrs 43.51 (11.27)	288	288	Nil	NCEP/ATP III (82), IDF (126)	NCEP/ATP III (28), IDF (44)	NCEP/ATP III and IDF
36	Yeboah et al., 2023 (71)	Ghana	West Africa	1 Health facility	Case-control	Male and female	Mean (SD) 38.6 (11.5)	450	300	150	118	39	JIS

IDF, International Diabetes Federation; NCEP/ATP III, National Cholesterol Education Program, Adult Treatment Panel; JIS, joint interim statement.

In terms of regional distribution, East Africa contributed the largest number of studies (18/36; 50.0%) (36, 37, 40–42, 47, 50–52, 55, 58–61, 65, 67, 69, 70), followed by West Africa (8/36; 22.2%) (45, 46, 48, 53, 62, 64, 68, 71), Southern Africa (7/36; 19.4%) (39, 43, 44, 49, 56, 63, 66), Central Africa (2/36; 5.6%) (54, 57), and North Africa (1/36; 2.8%) (38). Ethiopia was the most represented country (12/36; 33.3%) (36, 40–42, 47, 50–52, 61, 67, 69, 70), while South Africa contributed four studies (4/36; 11.1%) (39, 44, 63, 66), and Kenya (55, 59, 65), Nigeria (53, 62, 64), and Ghana (45, 68, 71) each contributed three studies (8.3%).

The included studies enrolled 17,101 participants, of which 15,191 were HIV-positive and 1,910 were HIV-negative. The median sample size across studies was 362 participants, ranging from 70 to 2,512. Nearly all studies included male and female (35/36; 97%) (36–65, 67–71), with one study focusing exclusively on female participants (66). Reporting of age was inconsistent, but where specified, participants ranged from 9 to 70 years, with most samples composed of adults in their 30s and 40s.

The diagnostic criteria for MetS varied considerably across the eligible studies (Table 2). Thirteen studies (36.1%) applied the International Diabetes Federation (IDF) definition (37, 38, 43, 44, 46–48, 52, 56–58, 60, 65), eight (22.2%) (45, 49, 51, 55, 61, 63, 68, 71) used the Joint Interim Statement (JIS), and six (16.7%) applied the National Cholesterol Education Program Adult Treatment Panel III (NCEP ATP III) criteria (40, 42, 53, 54, 62, 66). Seven studies (19.4%) used a combination of IDF and ATP III (36, 39, 50, 64, 67, 69, 70), one study (2.8%) combined IDF, ATP III, and JIS definitions (41), and one study (2.8%) applied the 2009 Consensus definition (59).

Among studies with sufficient data (*n* = 28), the median prevalence of MetS among PLHIV was 22.4%, with reported values ranging from 6.3% to 52.6%. Across all studies, a total of 4,234 cases of MetS in PLHIV were documented Table 3.

**Table 3:** Summary of study characteristics.

African region	Number of articles	Total number of HIV positive	Total number of metabolic syndrome	Prevalence of metabolic syndrome (%)	Mets criteria
Eastern Africa	18	5,972	1,523	25.5	IDF; *n* = 7 NCEP/ATP III; *n* = 8 JIS; *n* = 3
Western Africa	8	4,486	1,488	33.2	IDF; *n* = 2 NCEP/ATP III; *n* = 3 JIS; *n* = 3
Southern Africa	7	4,052	1,009	24.9	IDF; *n* = 3 NCEP/ATP III; *n* = 2 JIS; *n* = 2
Central Africa	2	611	195	31.9	IDF; *n* = 1 NCEP/ATP III; *n* = 1 JIS; *n* = 0
Northern Africa	1	70	19	27.1	IDF; *n* = 0 NCEP/ATP III; *n* = 1 JIS; *n* = 0
Total	36	15,191	4,234	27.9	

IDF, International Diabetes Federation; NCEP/ATP III, National Cholesterol Education Program, Adult Treatment Panel; JIS, joint interim statement.

### 3.3 Quality assessment of included studies

The risk of bias assessment, conducted using NOS, indicated that the majority of studies were of high methodological quality. Most studies (33/36; 92%) scored well across the three NOS domains; selection, comparability, and outcome assessment, suggesting generally low risk of bias (36, 47, 49–53, 55–57, 59–71).

The main weaknesses identified were limited reporting or justification of sample size in one study (38), inadequate control for potential confounding variables in five studies (36, 38, 48, 62, 70), and incomplete reporting of response rates or missing data in seven studies (36, 48, 51, 53, 59, 64, 65). Despite these limitations, 33 of the 36 studies achieved a NOS score of ≥7 and were classified as high quality (36, 47, 49–53, 55–57, 59–71). The remaining three studies scored between 4 and 6, placing them in the moderate-quality category (36, 38, 48). No studies were rated as low quality. Full scoring details for each study are presented in (Supplementary Table S6).

### 3.4 Meta-analysis

Of the 36 studies included in this review, 23 met the criteria for quantitative synthesis and were eligible for meta-analysis (37, 38, 40–43, 45–47, 49, 50, 52, 54–57, 60–63, 66, 67, 70), while 13 were excluded due to variation in outcome measures, inconsistent statistical reporting, or use of effect estimates that could not be harmonized (36, 39, 44, 48, 51, 53, 58, 59, 64, 65, 68, 69, 71). This ensured that only studies providing comparable data were pooled.

#### 3.4.1 Female sex

**Figure 2:**
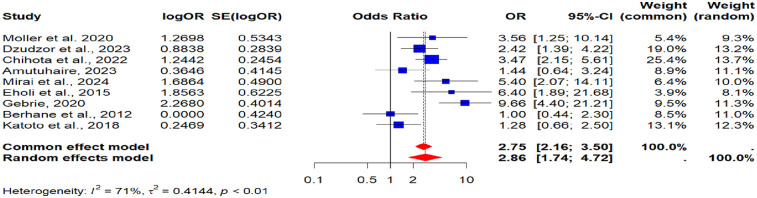
A forest plot showing the pooled odds ratio of the association between female sex and metabolic syndrome in people living with HIV.

Nine (9) studies contributed data on sex differences in the prevalence of MetS among PLHIV (37, 40, 43, 45–47, 54, 60, 61). The pooled analysis showed that being female was strongly associated with higher odds of MetS (POR = 2.86, 95% CI: 1.74–4.72). This indicates that women living with HIV have more than double the risk of developing MetS compared to men. Heterogeneity across these studies was moderate (*I*^2^ = 71%), reflecting some level of variability (see Figure 2).

#### 3.4.2 Alcohol consumption

**Figure 3:**
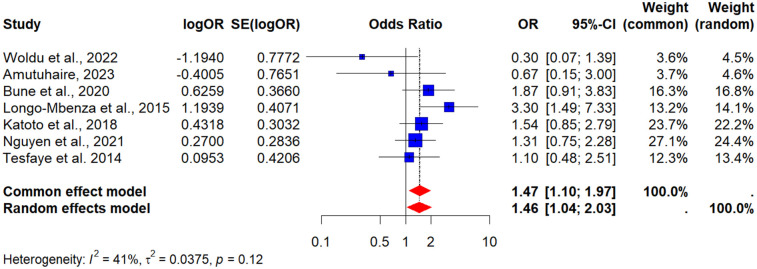
A forest plot showing the pooled odds ratio of the association between alcohol and metabolic syndrome in people living with HIV.

Seven (7) studies examined the impact of alcohol use (37, 42, 54, 57, 63, 67, 70). The PORs was 1.46 (95% CI: 1.04–2.03), showing that alcohol consumers had a 46% higher risk of developing MetS compared to non-drinkers. Heterogeneity was low (*I*^2^ = 41%), strengthening the reliability of this association (see Figure 3).

#### 3.4.3 Body mass index (BMI > 25 kg/m^2^)

**Figure 4:**
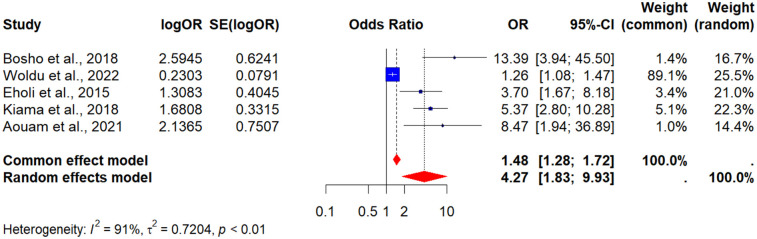
A forest plot showing the pooled odds ratio of the association between BMI > 25 kg/m^2^ and metabolic syndrome in people living with HIV.

Five (5) studies reported associations between BMI and MetS (38, 41, 46, 55, 70). Individuals with BMI above 25 had more than fourfold higher odds of developing MetS compared to those with lower BMI (POR = 4.27, 95% CI: 1.83–9.33). Heterogeneity was substantial (*I*^2^ = 91%), suggesting variability in study populations, ART regimens, or diagnostic criteria across included studies (see Figure 4).

#### 3.4.4 HIV status

**Figure 5:**
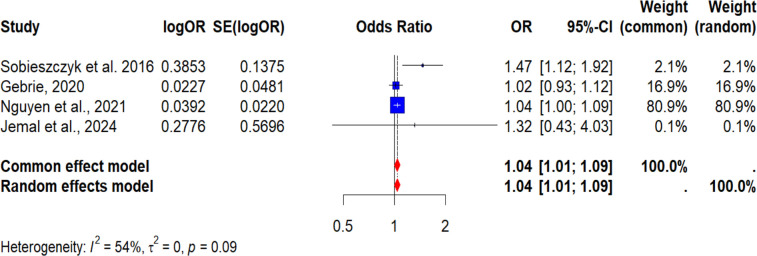
A forest plot showing the pooled odds ratio of the association between HIV and metabolic syndrome in people living with HIV.

The meta-analysis examining the influence of HIV status as a determinant of MetS in individuals living with HIV in Africa pooled data from four studies, yielding a combined OR of 1.04 (95% CI: 1.01, 1.09) with a moderate heterogeneity (*I*^2^ = 54%). This result indicates that individuals with HIV have a slightly elevated risk of developing MetS compared to those without HIV (see Figure 5).

#### 3.4.5 Smoking

**Figure 6:**
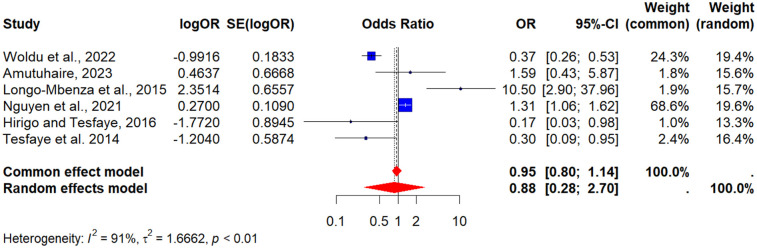
A forest plot showing the pooled odds ratio of the association between smoking and metabolic syndrome in people living with HIV.

Data from six (6) studies were pooled to assess smoking as a determinant (37, 50, 57, 63, 67, 70). The POR was 0.88 (95% CI: 0.48–2.70), indicating no significant association between smoking and MetS in PLHIV. Heterogeneity was very high (*I*^2^ = 91%), which may reflect differences in how smoking was measured, variations in prevalence across populations, and potential underreporting in some cohorts (see Figure 6).

#### 3.4.6 Physical activity

**Figure 7:**
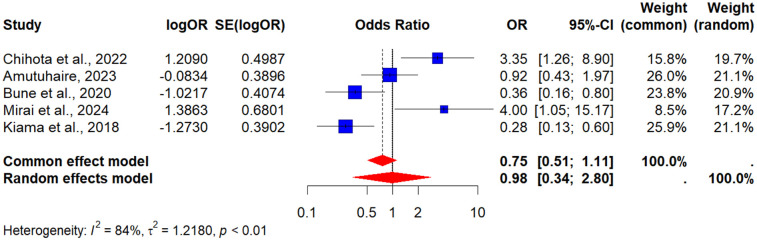
A forest plot showing the pooled odds ratio of the association between physical activity and metabolic syndrome in people living with HIV.

Five (5) studies assessed the role of physical activity (37, 42, 43, 55, 60). The POR was 0.98 (95% CI: 0.35–2.80), suggesting no significant association between physical activity and MetS in this population. Heterogeneity was substantial (*I*^2^ = 84%), likely due to differences in how activity levels were measured (self-report vs. objective measures) and the cross-sectional nature of most studies (see Figure 7).

### 3.5 Publication bias

Assessment of publication bias was performed for all six (6) determinants included in the meta-analysis. Visual inspection of funnel plots revealed asymmetry in several cases, suggesting the possibility of selective reporting or underrepresentation of smaller studies with null or negative findings (see Supplementary Figures S1a–f). To further investigate this, we applied the Trim-and-Fill method, which estimates the number of potentially missing studies and recalculates the pooled effect size accordingly. The adjusted results showed only minor differences compared to the original pooled odds ratios, indicating that while some publication bias may exist, its influence on the overall findings is likely limited (see Supplementary Figures S2a–f).

### 3.6 Sensitivity analysis

We conducted leave-one-out sensitivity analyses for each determinant to evaluate the robustness of our findings. The results demonstrated that the overall pooled effect sizes remained stable across all iterations, confirming that no single study exerted undue influence on the direction or magnitude of the associations (see Supplementary Figures S3a–f). This consistency across sensitivity analyses increases confidence in the reliability and validity of the pooled estimates.

### 3.7 GRADE assessment

Using the GRADE framework, the certainty of evidence varied across determinants of MetS among PLHIV in Africa. The association between female sex and MetS was rated as moderate certainty, downgraded for inconsistency due to moderate heterogeneity (*I*^2^ = 71%) but supported by a large effect size (POR = 2.86). Evidence for alcohol consumption as a risk factor was graded as moderate certainty, with consistent findings across studies (low heterogeneity, *I*^2^ = 41%) but some concerns for risk of bias in exposure measurement. Elevated BMI (>25) showed the strongest association (POR = 4.27), but the evidence was downgraded to low certainty due to very high heterogeneity (*I*^2^ = 91%) and variable measurement approaches across studies. The effect of HIV status was rated as low certainty, given the small effect size (OR = 1.04), moderate heterogeneity, and potential confounding. Smoking and physical activity were both graded as very low certainty, reflecting wide confidence intervals, substantial heterogeneity (*I*^2^ > 80%), and imprecision in measurement. Overall, the evidence base provides moderate certainty for sex and alcohol-related associations, low certainty for BMI and HIV status, and very low certainty for smoking and physical activity (see Supplementary Table S7).

## 4 Discussion

This systematic review and meta-analysis provide a comprehensive synthesis of the determinants of MetS among PLHIV in Africa. By pooling evidence from 36 studies conducted across multiple sub-regions, we identified female sex, elevated body BMI, and alcohol consumption as consistent risk factors for MetS, while smoking and physical activity showed no significant associations. These findings advance previous systematic reviews that primarily focused on prevalence (22, 72) or on global populations (18), by quantifying continent-specific determinants in African PLHIV and highlighting the unique interplay between biological, socio-cultural, and treatment-related factors.

### 4.1 Female sex and MetS

The strong association between female sex and MetS underscores the disproportionate vulnerability of women living with HIV in African settings. This finding is consistent with both regional evidence, such as an Ethiopian study reporting nearly double the risk among women (36) and broader sub-Saharan African trends that show higher cardiometabolic risk in women, particularly in urban areas (72). Globally, systematic reviews have also reported sex-based differences in MetS prevalence, although effect sizes are often smaller than those observed here (18).

Several mechanisms may underlie this disparity. Biological factors, including postmenopausal estrogen decline, higher body fat percentage, and differential fat distribution, likely contribute to this sex disparity (73–75). However, socio-cultural and structural influences in African settings likely amplify this effect. Disproportionate caregiving burdens, reduced opportunities for physical activity, and dietary shifts linked to urbanization have all been implicated (76, 77). Poor socioeconomic status further compounds this risk: women in Africa generally have lower economic power than men, limiting access to optimal treatment options and increasing vulnerability to metabolic complications.

The relationship between HIV and MetS itself also contributes to female predominance. HIV pathophysiology is associated with dyslipidemia, insulin resistance, and chronic inflammation, all of which drive metabolic syndrome. Women are more biologically and socially susceptible to HIV infection than men: the risk of HIV acquisition is estimated to be 70% higher in women, with male-to-female transmission being 2.3 times more efficient than female-to-male (78). It was also noted that women often sustain more health damage from HIV and other sexually transmitted infections due to economic, biological, and social vulnerabilities (79). As HIV itself is a determinant of MetS, this sex imbalance in infection contributes to the higher burden of MetS among women living with HIV in Africa.

Treatment-related factors are also critical. Women are often initiated on ART earlier and maintained longer, leading to higher cumulative exposure to metabolic effects (80–83). For instance, low- and middle-income countries are transitioning from NNRTI-based therapy to dolutegravir, an integrase strand transfer inhibitor. Although DTG generally has a favorable safety profile, emerging evidence from African cohorts suggests it is associated with weight gain and worse metabolic outcomes (53) (pl). Consequently, women living with HIV face heightened risks of abdominal obesity, dyslipidemia, dysglycemia, and hypertension, collectively explaining the female predominance of MetS in this population.

### 4.2 Alcohol consumption and MetS

Alcohol consumption was significantly associated with a higher risk of MetS among PLHIV, with a pooled odds ratio of 1.46, indicating a 46% increase in odds compared to non-drinkers. This finding is consistent with cohort data from African settings (84, 85) and aligns with earlier reviews linking alcohol use to HIV-related comorbidities (86). Unlike some studies in the general population, where moderate alcohol intake has been associated with favorable lipid profiles and cardioprotective effects (87), our results highlight the unique vulnerability of PLHIV. In this group, alcohol use may exacerbate ART-related metabolic toxicity, promote central adiposity, and reduce adherence to treatment regimens (88–90).

Importantly, the rising prevalence of hazardous drinking patterns documented in several African HIV cohorts (84, 91) underscores the urgency of addressing alcohol use as part of routine HIV care. Integrating targeted alcohol-reduction strategies, through counseling, behavioral interventions, and linkage to community-based support, could provide dual benefits by improving viral suppression and mitigating metabolic complications.

### 4.3 BMI and MetS

Elevated BMI emerged as the strongest determinant of MetS among PLHIV in Africa. This result is consistent with the global epidemiological consensus that overweight and obesity are principal drivers of MetS and cardiometabolic disease (92, 93). Within HIV populations, weight gain following ART initiation has been increasingly documented, particularly with integrase inhibitor–based regimens such as dolutegravir (94, 95). These changes may compound existing risk factors related to urbanization, shifting dietary patterns, and sedentary lifestyles in many African settings.

Despite this variability, the overall direction of the association underscores the urgent need to integrate lifestyle counselling and structured weight management into HIV care protocols. Early identification of patients at risk of obesity-related complications, alongside tailored interventions to promote a healthy diet and physical activity, may help reduce the burden of MetS in African PLHIV.

### 4.4 HIV status and MetS

The pooled analysis of HIV status as a determinant of MetS yielded a small but statistically significant effect, suggesting an increased risk among PLHIV compared to HIV-negative controls. This contrasts with findings from the pre-ART era, where HIV-related immune activation and chronic inflammation were considered dominant drivers of metabolic disturbances (96, 97). In the current ART era, where viral suppression is more widely achieved, these mechanisms may play a less central role.

It is increasingly plausible that the elevated risk of MetS in PLHIV is no longer attributable primarily to untreated HIV infection but rather to ART exposure and modifiable lifestyle factors, such as weight gain, diet, and physical inactivity. These findings reinforce the need to shift the clinical focus from HIV infection itself to the broader context of chronic disease management in ART-treated populations, integrating metabolic risk assessment into routine HIV care.

### 4.5 Smoking and physical activity

This review found no statistically significant association between either smoking or physical activity and the risk of MetS among PLHIV in Africa. However, these null findings should be interpreted with caution. For smoking, the wide confidence intervals may reflect socio-demographic, socio-economic, and cultural variability in smoking prevalence across African cohorts. Misclassification of smoking status and under-reporting could also have contributed to these results. In addition, some have suggested that the absence of association may relate to limited metabolic impact when tobacco products are incompletely metabolized. Supporting this, a South African study of nicotine metabolite ratios (NMR) among PLHIV reported minimal variability, raising questions about the clinical utility of smoking cessation biomarkers in this population (98). Similarly, there is a negative relationship between smoking and metabolic syndrome (98), highlighting the need for more nuanced evaluation of smoking-related risks in African contexts.

For physical activity, the absence of a significant effect may largely reflect inconsistencies in the measurement of exercise exposure across studies and the reliance on cross-sectional designs, which cannot adequately capture the long-term protective effects of regular exercise. Yet, previous African studies have shown that structured cardiorespiratory training can reduce waist circumference, lower body fat, and improve metabolic outcomes in PLHIV (43, 99).

Despite the lack of statistically significant findings in this review, both smoking cessation and physical activity promotion remain well-established cardiometabolic risk modifiers in the general population. Their integration into HIV care continues to be justified, not only for potential metabolic benefits but also for broader improvements in cardiovascular health and overall quality of life.

Taken together, our findings align with both regional and global trends. For instance, studies consistently show that women living with HIV face nearly double the risk of MetS compared to men, mirroring results from outside Africa (100, 101). This suggests that sex-based disparities in metabolic health are not context-bound but rather a global phenomenon with local amplifiers, such as limited access to preventive care and sociocultural dietary practices in African settings. Similarly, the strong association between elevated BMI and MetS reflects Africa's ongoing nutrition transition, compounded by ART-related weight gain, a pattern also observed in international HIV cohorts (102–104). Moreover, alcohol use, though culturally variable, emerges as a robust determinant in both African and global studies, reinforcing its dual role in undermining ART adherence and exacerbating metabolic risk. Accordingly, these findings shows the universality of certain MetS determinants, while also pointing to unique contextual drivers in African PLHIV populations.

### 4.6 Strengths and limitations

This review has several strengths, including a comprehensive multi-database search, adherence to PRISMA 2020 guidelines, predefined PROSPERO registration, and rigorous risk-of-bias assessment using the Newcastle–Ottawa Scale. The inclusion of studies from all African regions enhances generalizability, while meta-analytic synthesis provides more robust estimates than narrative summaries.

However, limitations should be noted. The predominance of cross-sectional designs limits causal inference, while substantial heterogeneity in some outcomes (e.g., BMI, smoking, physical activity) likely reflects differences in diagnostic criteria, measurement tools, and ART regimens. Sub-group analysis could not be performed due to low number of reports with consistent outcome. Evidence of publication bias suggests overrepresentation of significant findings. Additionally, few studies accounted for dietary factors, ART regimen type, or inflammatory markers, which may confound associations.

### 4.7 Implications for policy and practice

The consistent associations of female sex, alcohol use, and elevated BMI with MetS highlight the need for routine metabolic screening within HIV care, particularly for women and overweight patients. Integrated interventions, including nutritional counselling, alcohol reduction, and physical activity promotion, should be embedded in ART programs. ART regimen choice should also consider metabolic risk profiles, especially in high-BMI patients, and integrated HIV–NCD platforms may improve the cost-effectiveness of service delivery.

### 4.8 Future research

Longitudinal cohort studies are needed to establish causal pathways and capture dynamic metabolic changes over time. Standardization of diagnostic criteria and measurement protocols will improve comparability across settings. Research should further examine sex-specific mechanisms, ART-related weight gain, and gene–environment interactions. Finally, culturally tailored lifestyle interventions and cost-effectiveness studies will be critical for informing sustainable integration of metabolic care into HIV programs.

## 5 Conclusion

This systematic review and meta-analysis demonstrate that MetS among PLHIV in Africa is influenced by multiple determinants, with female sex, alcohol consumption, and elevated BMI identified as the most consistent risk factors. These findings emphasize the urgent need to integrate routine metabolic screening and targeted prevention into HIV care, supported by gender-sensitive, lifestyle-focused, and context-specific interventions. Strengthening HIV–NCD integration within health systems will be critical to reducing the growing cardiometabolic burden in African PLHIV populations.

## Data Availability

The original contributions presented in the study are included in the article/Supplementary Material, further inquiries can be directed to the corresponding author.
