# Supplementary material for: Determinants of metabolic syndrome in people living with human immunodeficiency virus in Africa: a systematic review and meta-analysis
Source: Front Reprod Health. 2025 Nov 4;7:1689731. doi: 10.3389/frph.2025.1689731 (PMC12623360; doi:10.3389/frph.2025.1689731)
Supplement: Supplementary file 1 [file Datasheet1.zip › Supplementary Files.docx]

**Supplementary Files**

**Table S2: SEARCH STRATEGY**

**PubMed Search Strategy**

| S/N | Keywords | Search Strategy | Results / Time |
| --- | --- | --- | --- |
| #1 | Determinants | "determinants"[Title/Abstract] OR "factor*"[Title/Abstract] OR "Predictors"[Title/Abstract] OR "Risk factors"[MeSH Terms] OR "Risk factors"[Title/Abstract] OR "Epidemiology"[Title/Abstract] OR "Epidemiology"[MeSH Terms] OR "Predisposing factors"[Title/Abstract] | [5,764,717](https://pubmed.ncbi.nlm.nih.gov/?term=%22determinants%22%5BTitle%2FAbstract%5D+OR+%22factor%2A%22%5BTitle%2FAbstract%5D+OR+%22Predictors%22%5BTitle%2FAbstract%5D+OR+%22Risk+factors%22%5BMeSH+Terms%5D+OR+%22Risk+factors%22%5BTitle%2FAbstract%5D+OR+%22Epidemiology%22%5BTitle%2FAbstract%5D+OR+%22Epidemiology%22%5BMeSH+Terms%5D+OR+%22Predisposing+factors%22%5BTitle%2FAbstract%5D&ac=no&sort=relevance)  16:08:33 |
| #2 | Metabolic syndrome | "Metabolic syndrome"[MeSH Terms] OR "Metabolic syndrome"[Title/Abstract] OR "dysmetabolic syndrome"[Title/Abstract] OR "cardiometabolic syndrome"[Title/Abstract] OR "syndrome x"[Title/Abstract] OR "deadly quartet"[Title/Abstract] OR "Reaven's syndrome"[Title/Abstract] OR "metabolic Cardiovascular Syndrome"[Title/Abstract] OR "insulin resistance syndrome"[Title/Abstract] OR "MetS"[Title/Abstract] | [85,430](https://pubmed.ncbi.nlm.nih.gov/?term=%22Metabolic+syndrome%22%5BMeSH+Terms%5D+OR+%22Metabolic+syndrome%22%5BTitle%2FAbstract%5D+OR+%22dysmetabolic+syndrome%22%5BTitle%2FAbstract%5D+OR+%22cardiometabolic+syndrome%22%5BTitle%2FAbstract%5D+OR+%22syndrome+x%22%5BTitle%2FAbstract%5D+OR+%22deadly+quartet%22%5BTitle%2FAbstract%5D+OR+%22Reaven%27s+syndrome%22%5BTitle%2FAbstract%5D+OR+%22metabolic+Cardiovascular+Syndrome%22%5BTitle%2FAbstract%5D+OR+%22insulin+resistance+syndrome%22%5BTitle%2FAbstract%5D+OR+%22MetS%22%5BTitle%2FAbstract%5D&ac=no&sort=relevance)  16:15:56 |
| #3 | PLWH | "HIV"[Mesh Terms] OR "PLWH"[Title/Abstract] OR "PLHIV"[Title/Abstract] OR "human immunodeficiency virus"[Title/Abstract] OR "human immunedeficiency virus"[Title/Abstract] OR "AIDS Virus*"[Title/Abstract] OR "AIDS"[Title/Abstract] OR "HIV"[Title/Abstract] OR "HIV-1"[Mesh Terms] OR "HIV-1"[Title/Abstract] OR "HIV-2"[Mesh Terms] OR "HIV-2"[Title/Abstract] OR "HIV-positive"[Title/Abstract] OR "HIV-infected"[Title/Abstract] OR "acquired immunodeficiency syndrome"[Title/Abstract] OR "acquired immunodeficiency syndrome"[Mesh Terms] | [510,195](https://pubmed.ncbi.nlm.nih.gov/?term=%22HIV%22%5BMesh+Terms%5D+OR+%22PLWH%22%5BTitle%2FAbstract%5D+OR+%22PLHIV%22%5BTitle%2FAbstract%5D+OR+%22human+immunodeficiency+virus%22%5BTitle%2FAbstract%5D+OR+%22human+immunedeficiency+virus%22%5BTitle%2FAbstract%5D+OR+%22AIDS+Virus%2A%22%5BTitle%2FAbstract%5D+OR+%22AIDS%22%5BTitle%2FAbstract%5D+OR+%22HIV%22%5BTitle%2FAbstract%5D+OR+%22HIV-1%22%5BMesh+Terms%5D+OR+%22HIV-1%22%5BTitle%2FAbstract%5D+OR+%22HIV-2%22%5BMesh+Terms%5D+OR+%22HIV-2%22%5BTitle%2FAbstract%5D+OR+%22HIV-positive%22%5BTitle%2FAbstract%5D+OR+%22HIV-infected%22%5BTitle%2FAbstract%5D+OR+%22acquired+immunodeficiency+syndrome%22%5BTitle%2FAbstract%5D+OR+%22acquired+immunodeficiency+syndrome%22%5BMesh+Terms%5D&ac=no&sort=relevance)  16:17:32 |
| #4 | Africa | (((("Africa"[MeSH Terms] OR "Africa"[Title/Abstract] OR "South Africa"[Title/Abstract] OR "Southern Africa"[Title/Abstract] OR "Western Africa"[Title/Abstract] OR "West Africa"[Title/Abstract] OR "Northern Africa"[Title/Abstract] OR "North Africa"[Title/Abstract] OR "Eastern Africa"[Title/Abstract] OR "East Africa"[Title/Abstract] OR "Central Africa"[Title/Abstract] OR "Algeria"[Title/Abstract] OR "Egypt"[Title/Abstract] OR "Libya"[Title/Abstract] OR "Mauritania"[Title/Abstract] OR "Morrocco"[Title/Abstract] OR "Tunisia"[Title/Abstract] OR "Benin"[Title/Abstract] OR "Burkina Faso"[Title/Abstract] OR "Cape Verde"[Title/Abstract] OR "Cote d'Ivoire"[Title/Abstract] OR "Gambia"[Title/Abstract] OR "Ghana"[Title/Abstract] OR "Guinea-Bissau"[Title/Abstract] OR "Guinea"[Title/Abstract] OR "Liberia"[Title/Abstract] OR "Mali"[Title/Abstract] OR "Senegal"[Title/Abstract] OR "Niger"[Title/Abstract] OR "Nigeria"[Title/Abstract] OR "sierra leone"[Title/Abstract] OR "Togo"[Title/Abstract] OR "Burundi"[Title/Abstract] OR "Comoros"[Title/Abstract] OR "Djibouti"[Title/Abstract] OR "Eritrea"[Title/Abstract] OR "Ethiopia"[Title/Abstract] OR "Kenya"[Title/Abstract] OR "Madagascar"[Title/Abstract] OR "Malawi"[Title/Abstract] OR "Mauritius"[Title/Abstract] OR "Mozambique"[Title/Abstract] OR "Rwanda"[Title/Abstract] OR "Seychelles"[Title/Abstract] OR "Somalia"[Title/Abstract] OR "south sudan"[Title/Abstract] OR "Tanzania"[Title/Abstract] OR "Uganda"[Title/Abstract] OR "Zambia"[Title/Abstract] OR "Zimbabwe"[Title/Abstract] OR "Angola"[Title/Abstract] OR "Cameroon"[Title/Abstract] OR "Central African Republic"[Title/Abstract] OR "Chad"[Title/Abstract] OR "Republic of the Congo"[Title/Abstract] OR "Democratic Republic of the Congo"[Title/Abstract] OR "Equatorial Guinea"[Title/Abstract] OR "Gabon"[Title/Abstract] OR "Botswana"[Title/Abstract] OR "Lesotho"[Title/Abstract] OR "Namibia"[Title/Abstract] OR "Swaziland"[Title/Abstract] OR "sub-Sahara Africa"[Title/Abstract] OR "Eswatini"[Title/Abstract]) OR ("Cabo Verde"[Title/Abstract])) OR ("Ivory Coast"[Title/Abstract])) OR ("DR Congo"[Title/Abstract])) OR ("Sao Tome and Principe"[Title/Abstract]) | [621,833](https://pubmed.ncbi.nlm.nih.gov/?term=longquery250a803d82696725e991&ac=no&sort=relevance)  16:18:57 |
| #5 |  | (((#1) AND (#2)) AND (#3)) AND (#4) | [89](https://pubmed.ncbi.nlm.nih.gov/?term=%28%28%28%231%29+AND+%28%232%29%29+AND+%28%233%29%29+AND+%28%234%29&ac=no&sort=relevance)  16:22:19 |
| #6 |  | (((#1) AND (#2)) AND (#3)) AND (#4) Filters: Exclude preprints | [88](https://pubmed.ncbi.nlm.nih.gov/?term=%28%28%28%231%29+AND+%28%232%29%29+AND+%28%233%29%29+AND+%28%234%29&filter=other.excludepreprints&ac=no&sort=relevance)  16:29:52 |

**Web of Science Search Strategy**

| S/N | Keywords | Search Strategy | Results |
| --- | --- | --- | --- |
| #1 | Determinants | (((((TS=(Determinants)) OR TS=(factor*)) OR TS=(predictors)) OR TS=("risk factors")) OR TS=(epidemiology)) OR TS=("Predisposing factors") | [9,105,009](https://www-webofscience-com.proxy.kib.ki.se/wos/woscc/summary/8ec2c03d-6d95-44ab-bea7-c8bde952df68-0169938c1a/relevance/1) |
| #2 | Metabolic syndrome | ((((((((TS=("Metabolic syndrome")) OR TS=("dysmetabolic syndrome")) OR TS=("cardiometabolic syndrome")) OR TS=("syndrome X")) OR TS=("deadly quartet")) OR TS=("Reaven’s syndrome")) OR TS=("metabolic cardiovascular syndrome")) OR TS=(MetS)) OR TS=("insulin resistance syndrome") | [140,694](https://www-webofscience-com.proxy.kib.ki.se/wos/woscc/summary/b18f24d5-2ff1-4d97-a91f-fc7b960b2de9-016993af8d/relevance/1) |
| #3 | PLWH | (((((((((((TS=(PLWH)) OR TS=(PLHIV)) OR TS=("Human Immunodeficiency Virus")) OR TS=("Human Immunedeficiency Virus")) OR TS=("AIDS Virus")) OR TS=(AIDS)) OR TS=(HIV)) OR TS=(HIV-1)) OR TS=(HIV-2)) OR TS=(HIV-positive)) OR TS=(HIV-infected)) OR TS=("acquired immunodeficiency syndrome") | [1,161,472](https://www-webofscience-com.proxy.kib.ki.se/wos/woscc/summary/32539e17-6285-45dc-b647-44a7b4c5b314-016993c9a6/relevance/1) |
| #4 |  | #1 AND #2 AND #3 | [1,238](https://www-webofscience-com.proxy.kib.ki.se/wos/woscc/summary/b21c0b02-d743-4dfa-ac1a-5000982207a6-016993d5b1/relevance/1) |
| #5 |  | #1 AND #2 AND #3 and SOUTH AFRICA or ETHIOPIA or UGANDA or NIGERIA or TANZANIA or CAMEROON or GHANA or KENYA or ZAMBIA or DEM REP CONGO or SUDAN or BOTSWANA or MOZAMBIQUE or SENEGAL or ZIMBABWE or BENIN or NAMIBIA or REP CONGO or ALGERIA or BURKINA FASO or COTE IVOIRE or EGYPT or ESWATINI or GUINEA BISSAU or MOROCCO or RWANDA or TUNISIA (Countries/Regions) | [180](https://www-webofscience-com.proxy.kib.ki.se/wos/woscc/summary/64cf02ab-d638-4141-aaf0-8d55cffcbb7d-016993fd51/relevance/1) |
|  |  |  |  |

**CINAHL Search Strategy**

| S/N | Keywords | Search Strategy | Results |
| --- | --- | --- | --- |
| #1 | Determinants | XB determinants OR XB factor* OR XB predictors OR XB “risk factors” OR XB epidemiology OR XB “Predisposing factors" | View Results (1,059,055) |
| #2 | Metabolic syndrome | XB “metabolic syndrome” OR XB “dysmetabolic syndrome” OR XB “cardiometabolic syndrome” OR XB “syndrome X” OR XB “deadly quartet” OR XB “Reaven’s syndrome” OR XB “metabolic cardiovascular syndrome” OR XB MetS OR "insulin resistance syndrome" | View Results (117,240) |
| #3 | PLWH | XB PLWH OR XB PLHIV OR XB “Human Immunodeficiency Virus" OR XB “Human Immunedeficiency Virus” OR XB AIDS Virus OR XB AIDS OR XB HIV OR XB HIV-1 OR XB HIV-2 OR XB HIV-positive OR XB HIV-infected OR XB “acquired immunodeficiency syndrome” | View Results (141,056) |
| #4 | S1 AND S2 AND S3 |  | View Results (748) |
| #5 | S1 AND S2 AND S3 | Limiters - Publication Date: 20000101-20251231  Expanders - Apply equivalent subjects  Search modes - Find all my search terms | View Results (726) |

**SCOPUS Search Strategy**

| S/N | KEYWORDS | SEARCH STRATEGY | RESULTS |
| --- | --- | --- | --- |
| #1 | Determinants | determinants OR factor* OR predictors OR "risk factors" OR epidemiology OR "predisposing factors" | 14,024,593 |
| #2 | Metabolic syndrome | "Metabolic syndrome" OR "dysmetabolic syndrome" OR "cardiometabolic syndrome" OR "syndrome x" OR "deadly quartet" OR "Reaven's syndrome" OR "metabolic Cardiovascular Syndrome" OR "insulin resistance syndrome" OR Mets | 125,698 |
| #3 | PLWHS/N Keywords Search Strategy Results  #1 Determinants (((((TS=(determinant)) OR TS=(factor*)) OR TS=(predictors)) OR TS=("risk factors")) OR TS=(epidemiology)) OR TS=("Predisposing factors" ) 9,105,009  #2 Metabolic syndrome ((((((((TS=("Metabolic syndrome")) OR TS=("dysmetabolic syndrome" )) OR TS=("cardiometabolic syndrome")) OR TS=("syndrome X")) OR TS=("deadly quartet")) OR TS=("Reaven’s syndrome")) OR TS=("metabolic cardiovascular syndrome")) OR TS=(MetS)) OR TS=("insulin resistance syndrome") 140,694  #3 PLWH (((((((((((TS=(PLWH)) OR TS=(PLHIV)) OR TS=("Human Immunodeficiency Virus")) OR TS=("Human Immunedeficiency Virus")) OR TS=("AIDS Virus")) OR TS=(AIDS)) OR TS=(HIV)) OR TS=(HIV-1)) OR TS=(HIV-2)) OR TS=(HIV-positive)) OR TS=(HIV-infected)) OR TS=("acquired immunodeficiency syndrome") 1,161,472  #4 #1 AND #2 AND #3 1,238  #5 #4 FILTER BY COUNTRY 180  WEB OF SCIENCE | HIV OR PLWH OR PLHIV OR "human immunodeficiency virus" OR "human immunedeficiency virus" OR "AIDS Virus*" OR AIDS OR "HIV-1" OR "HIV-2" OR "HIV-positive" OR "HIV-infected" OR "acquired immunodeficiency syndrome" | 795,786 |
| #4 | #1 AND #2 AND #3 |  | 1,106 |
| #5 | #4 FILTER BY YEAR |  | 1,106 |
| #6 | #5 FILTER BY COUNTRIES |  | 153 |

Table S6: Bias risk by domains of quality assessment characteristics of included studies for determinants of metabolic syndrome in people living with HIV in Africa

| S/N | Selection |  |  |  | Comparability | Outcome |  |  | Total score | Study  Quality |
| --- | --- | --- | --- | --- | --- | --- | --- | --- | --- | --- |
|  | Representativeness of the exposed cohort | Sample size justification | Ascertainment of exposure | Non respondent or missing data | Control for Confounders | Assessment of outcome | Appropriate statistical tool for outcome | Reliability of the outcome |  |  |
| 1 | 1 | 1 | 1 | 1 | 2 | 1 | 1 | 1 | 9 | High |
| 2 | 1 | 1 | 0 | 1 | 2 | 1 | 1 | 1 | 8 | High |
| 3 | 1 | 1 | 1 | 1 | 2 | 1 | 1 | 1 | 9 | High |
| 4 | 1 | 1 | 1 | 0 | 2 | 1 | 1 | 1 | 8 | High |
| 5 | 1 | 1 | 1 | 1 | 1 | 1 | 1 | 1 | 8 | High |
| 6 | 1 | 1 | 1 | 1 | 2 | 1 | 1 | 1 | 9 | High |
| 7 | 1 | 1 | 1 | 1 | 2 | 1 | 1 | 1 | 9 | High |
| 8 | 1 | 1 | 1 | 1 | 2 | 1 | 1 | 1 | 9 | High |
| 9 | 1 | 1 | 1 | 1 | 2 | 1 | 1 | 1 | 9 | High |
| 10 | 1 | 1 | 1 | 0 | 2 | 1 | 1 | 1 | 8 | High |
| 11 | 1 | 1 | 1 | 1 | 2 | 1 | 1 | 1 | 9 | High |
| 12 | 1 | 1 | 1 | 1 | 0 | 1 | 1 | 1 | 7 | High |
| 13 | 1 | 1 | 1 | 0 | 0 | 1 | 1 | 1 | 6 | Moderate |
| 14 | 1 | 1 | 1 | 0 | 0 | 1 | 1 | 1 | 6 | Moderate |
| 15 | 1 | 1 | 1 | 0 | 2 | 1 | 1 | 1 | 8 | High |
| 16 | 1 | 1 | 1 | 0 | 2 | 1 | 1 | 1 | 8 | High |
| 17 | 1 | 1 | 1 | 0 | 2 | 1 | 1 | 1 | 8 | High |
| 18 | 1 | 1 | 1 | 1 | 2 | 1 | 1 | 1 | 9 | High |
| 19 | 1 | 1 | 1 | 1 | 2 | 1 | 1 | 1 | 9 | High |
| 20 | 1 | 1 | 1 | 1 | 2 | 1 | 1 | 1 | 9 | High |
| 21 | 1 | 1 | 1 | 1 | 2 | 1 | 1 | 1 | 9 | High |
| 22 | 1 | 1 | 1 | 1 | 2 | 1 | 1 | 1 | 9 | High |
| 23 | 1 | 1 | 1 | 1 | 2 | 1 | 1 | 1 | 9 | High |
| 24 | 1 | 1 | 1 | 1 | 2 | 1 | 1 | 1 | 9 | High |
| 25 | 1 | 0 | 1 | 1 | 0 | 1 | 1 | 1 | 6 | Moderate |
| 26 | 1 | 1 | 1 | 1 | 2 | 1 | 1 | 1 | 9 | High |
| 27 | 1 | 1 | 1 | 1 | 2 | 1 | 1 | 1 | 9 | High |
| 28 | 1 | 1 | 1 | 1 | 2 | 1 | 1 | 1 | 9 | High |
| 29 | 1 | 1 | 1 | 1 | 2 | 1 | 1 | 1 | 9 | High |
| 30 | 1 | 1 | 1 | 1 | 2 | 1 | 1 | 1 | 9 | High |
| 31 | 1 | 1 | 0 | 1 | 2 | 0 | 0 | 0 | 7 | High |
| 32 | 1 | 1 | 1 | 1 | 2 | 1 | 1 | 1 | 9 | High |
| 33 | 1 | 1 | 1 | 1 | 2 | 0 | 1 | 1 | 8 | High |
| 34 | 1 | 1 | 1 | 1 | 2 | 1 | 1 | 1 | 9 | High |
| 35 | 1 | 1 | 1 | 1 | 2 | 1 | 1 | 1 | 9 | High |
| 36 | 1 | 1 | 1 | 1 | 2 | 1 | 1 | 1 | 9 | High |

**Table S7: GRADE ASSESSMENT; Summary of Findings Table**

| **Determinant** | **Effect  (Pooled OR, 95% CI)** | **No. of studies** | **Certainty of evidence (GRADE)** | **Reasons for rating** |
| --- | --- | --- | --- | --- |
| **Female sex** | **OR = 2.86 (1.74–4.72)** | **9 studies** | **⬤⬤⬤◯ Moderate** | **Downgraded for inconsistency (I²=71%); upgraded for large effect** |
| **Alcohol consumption** | **OR = 1.46 (1.04–2.03)** | **5 studies** | **⬤⬤⬤◯ Moderate** | **Downgraded for risk of bias in exposure measurement; consistent effect** |
| **BMI >25** | **OR = 4.27 (1.83–9.33)** | **5 studies** | **⬤⬤◯◯ Low** | **Downgraded for very high heterogeneity (I²=91%); upgraded for strong effect** |
| **HIV status** | **OR = 1.04 (1.01–1.09)** | **4 studies** | **⬤⬤◯◯ Low** | **Downgraded for imprecision and moderate heterogeneity (I²=54%)** |
| **Smoking** | **OR = 0.88 (0.48–2.70)** | **5 studies** | **⬤◯◯◯ Very low** | **Downgraded for wide CI, imprecision, and high heterogeneity (I²=91%)** |
| **Physical activity** | **OR = 0.98 (0.35–2.80)** | **5 studies** | **⬤◯◯◯ Very low** | **Downgraded for imprecision and inconsistency (I²=84%)** |

**⬤⬤⬤⬤ = High;**

**⬤⬤⬤◯ = Moderate;**

**⬤⬤◯◯ = Low;**

**⬤◯◯◯ = Very low**


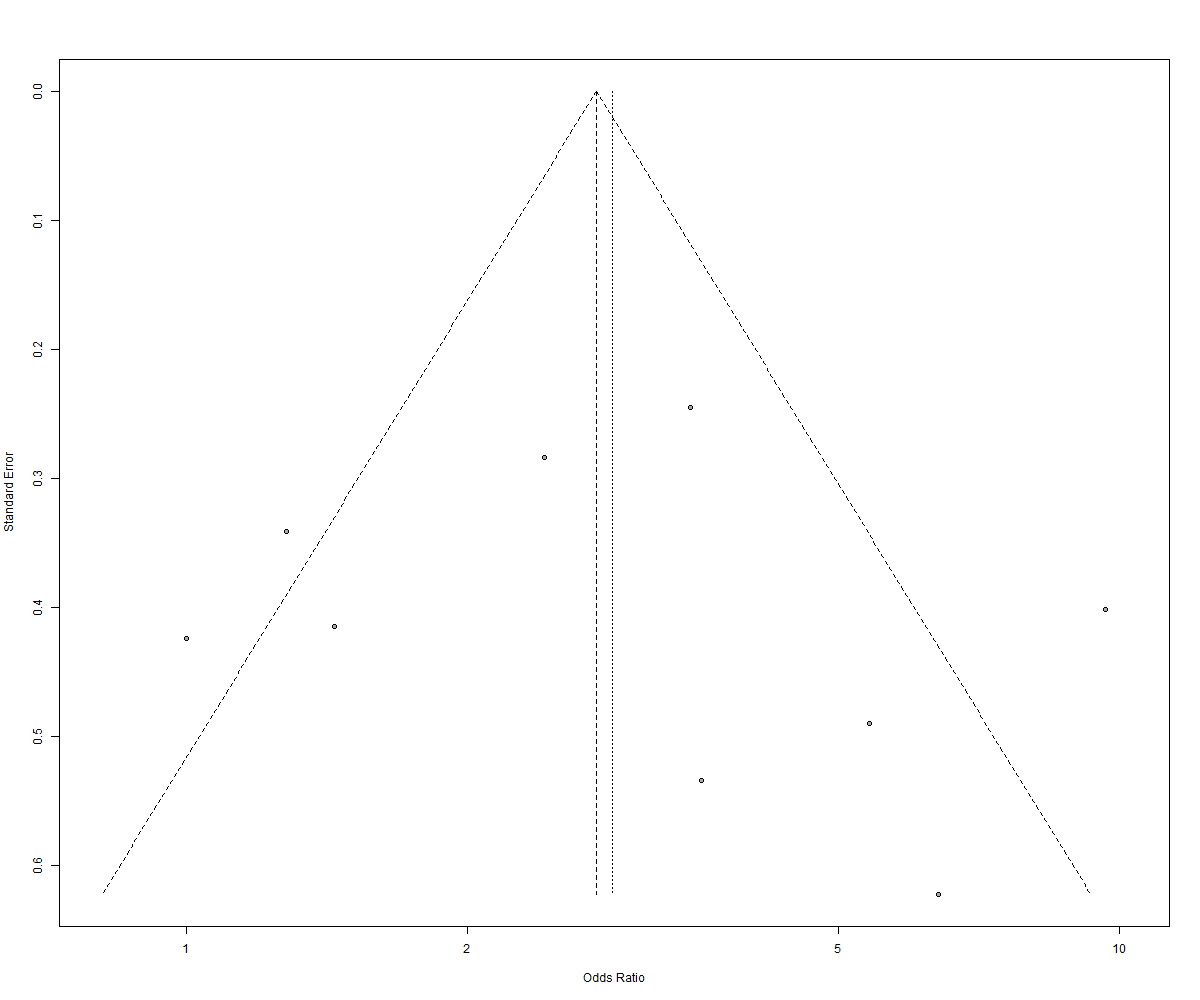

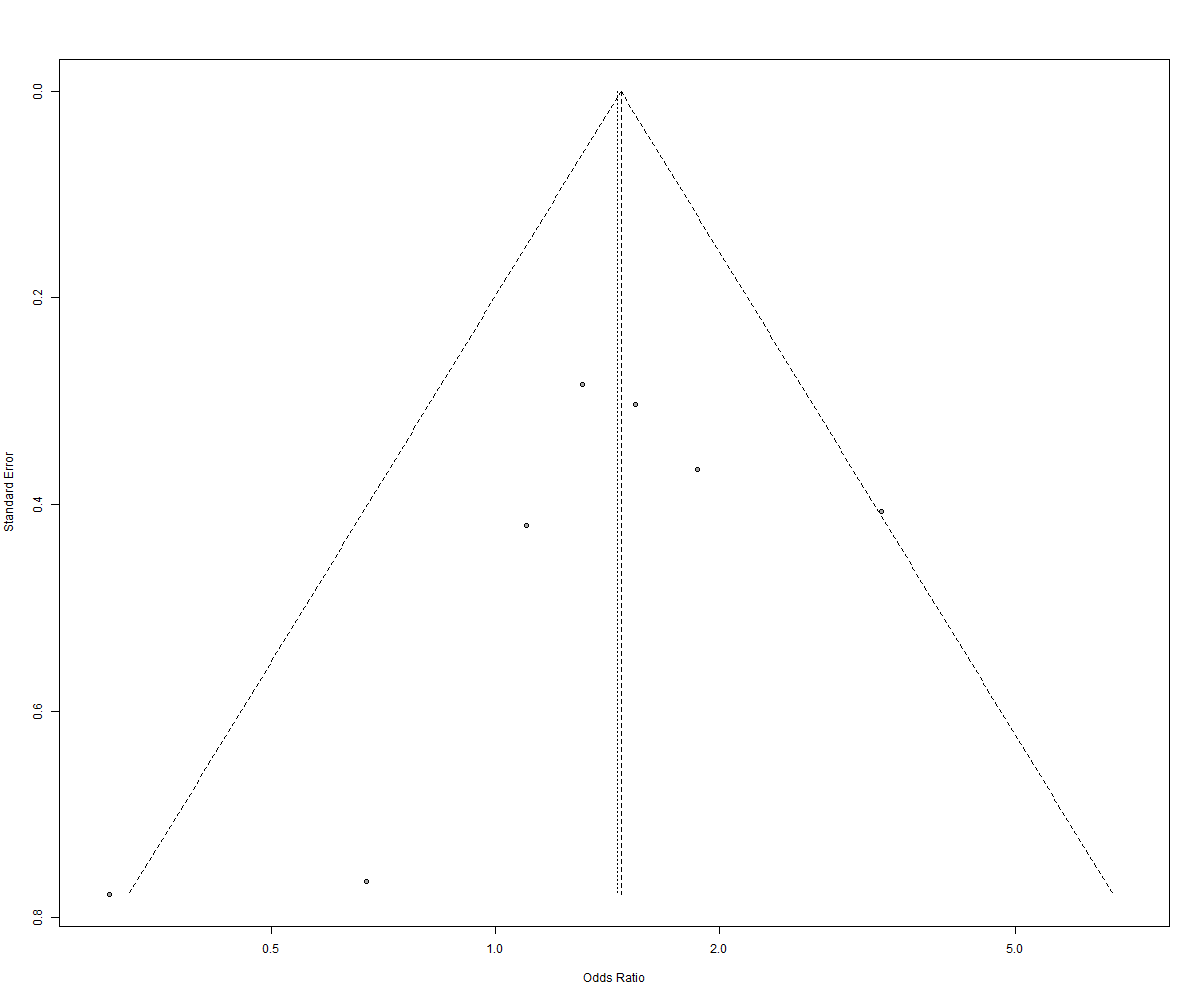


Figure S1a: Female sex Figure S1b: Alcohol


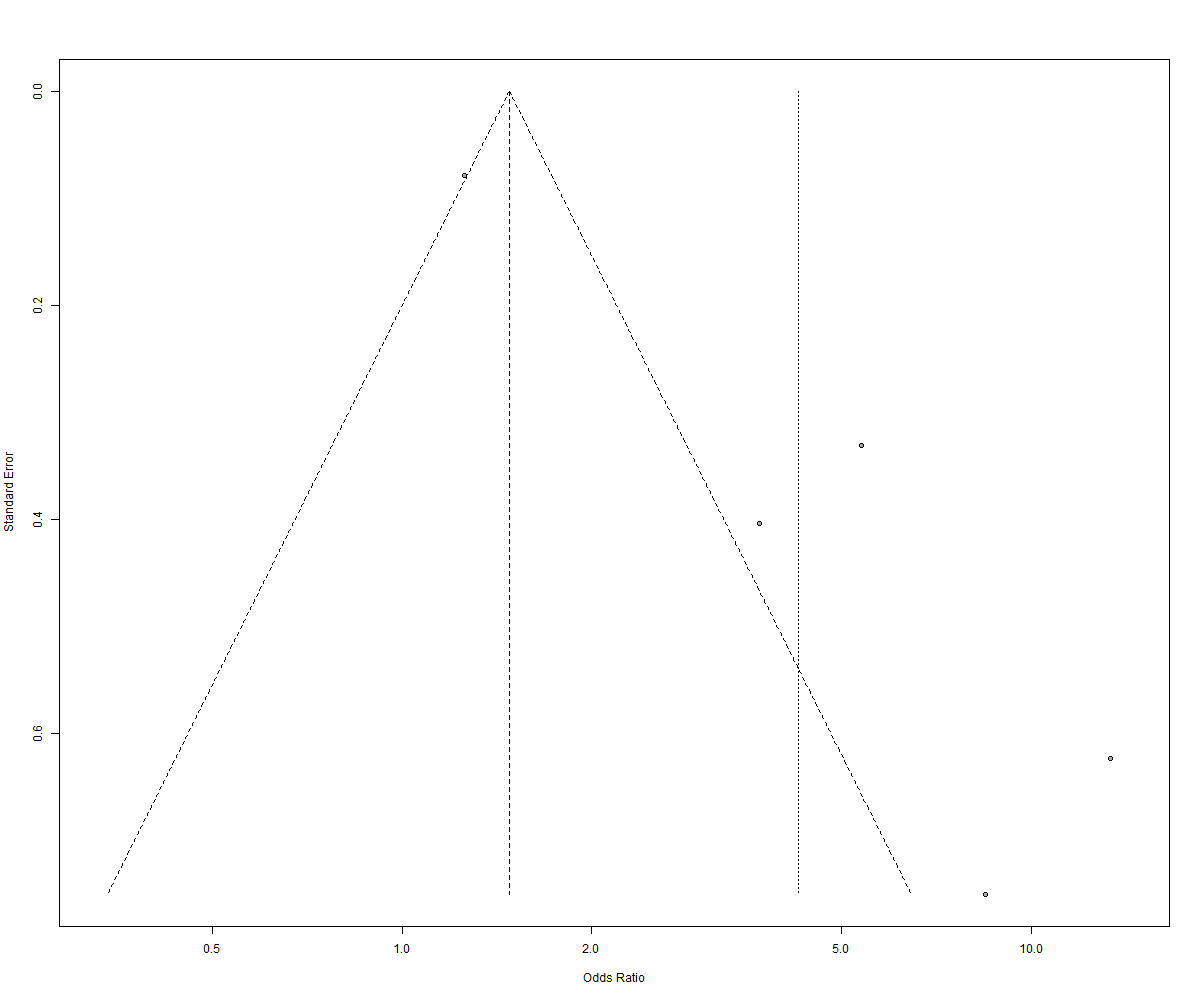

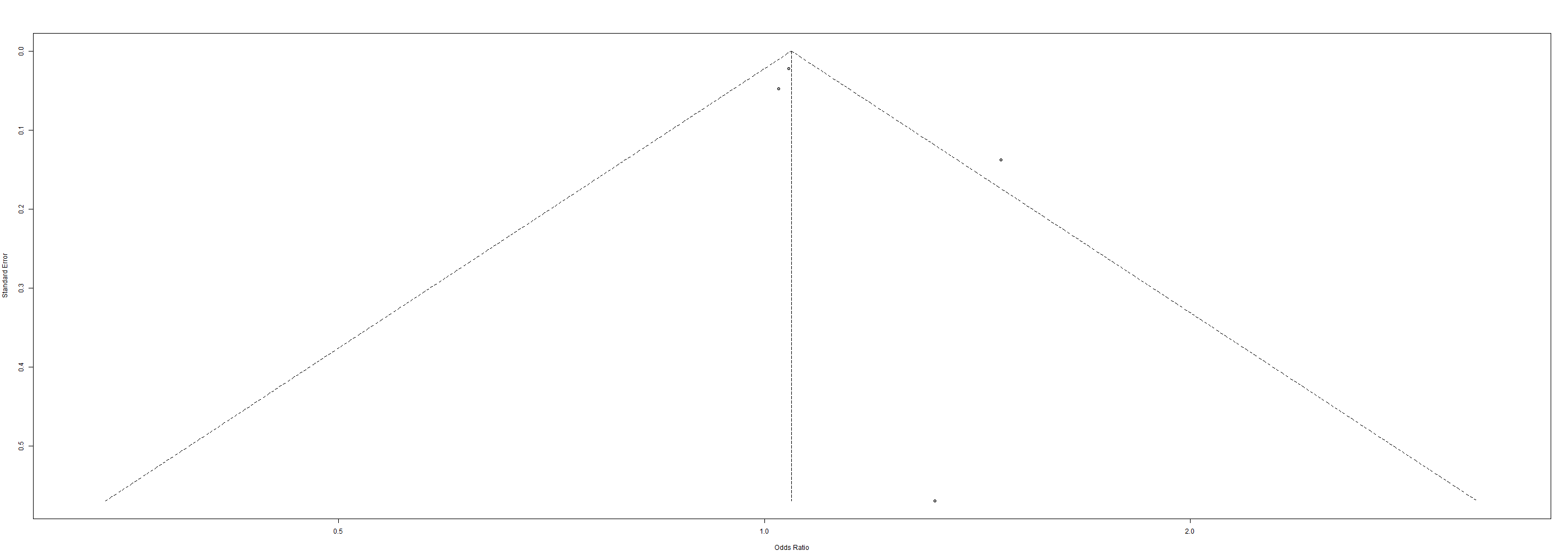


Figure S1c: BMI> 25kg/ m^2^ Figure S1d: HIV


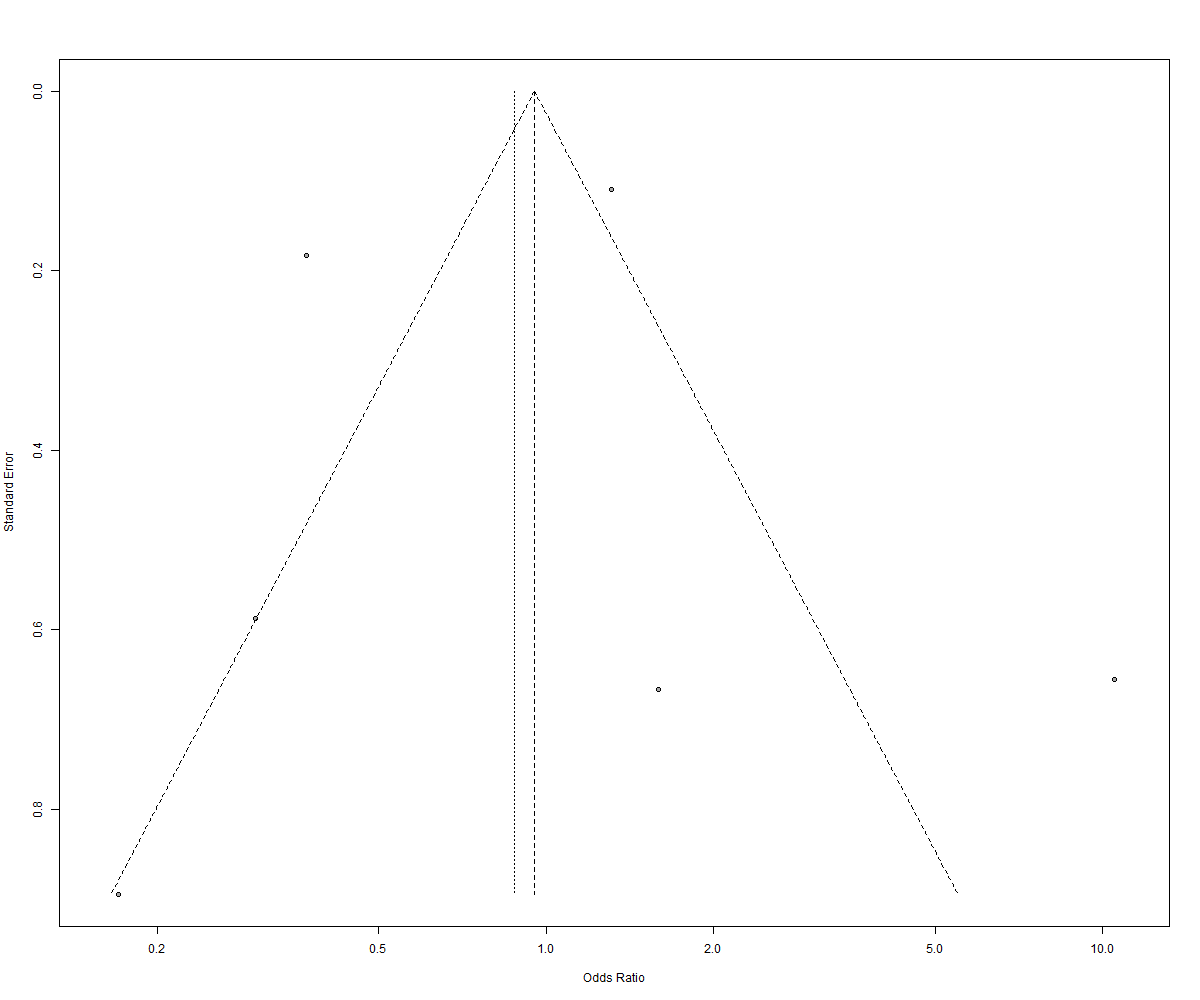

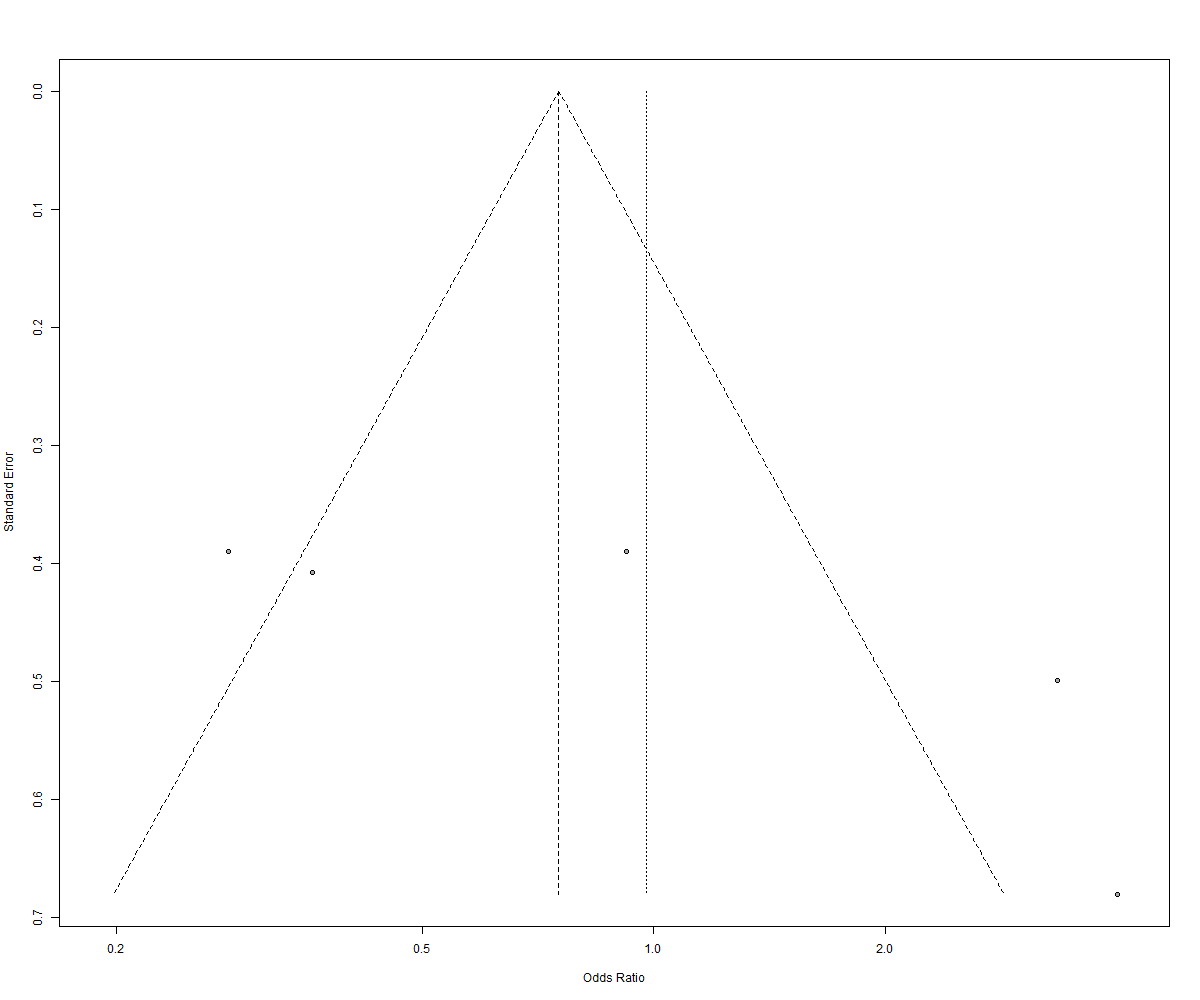


Figure S1e: Smoking Figure S1f: Physical activity

Figure S1a – S1f: Funnel plots for assessment of publication bias for all six determinants of metabolic syndrome included in the meta-analysis


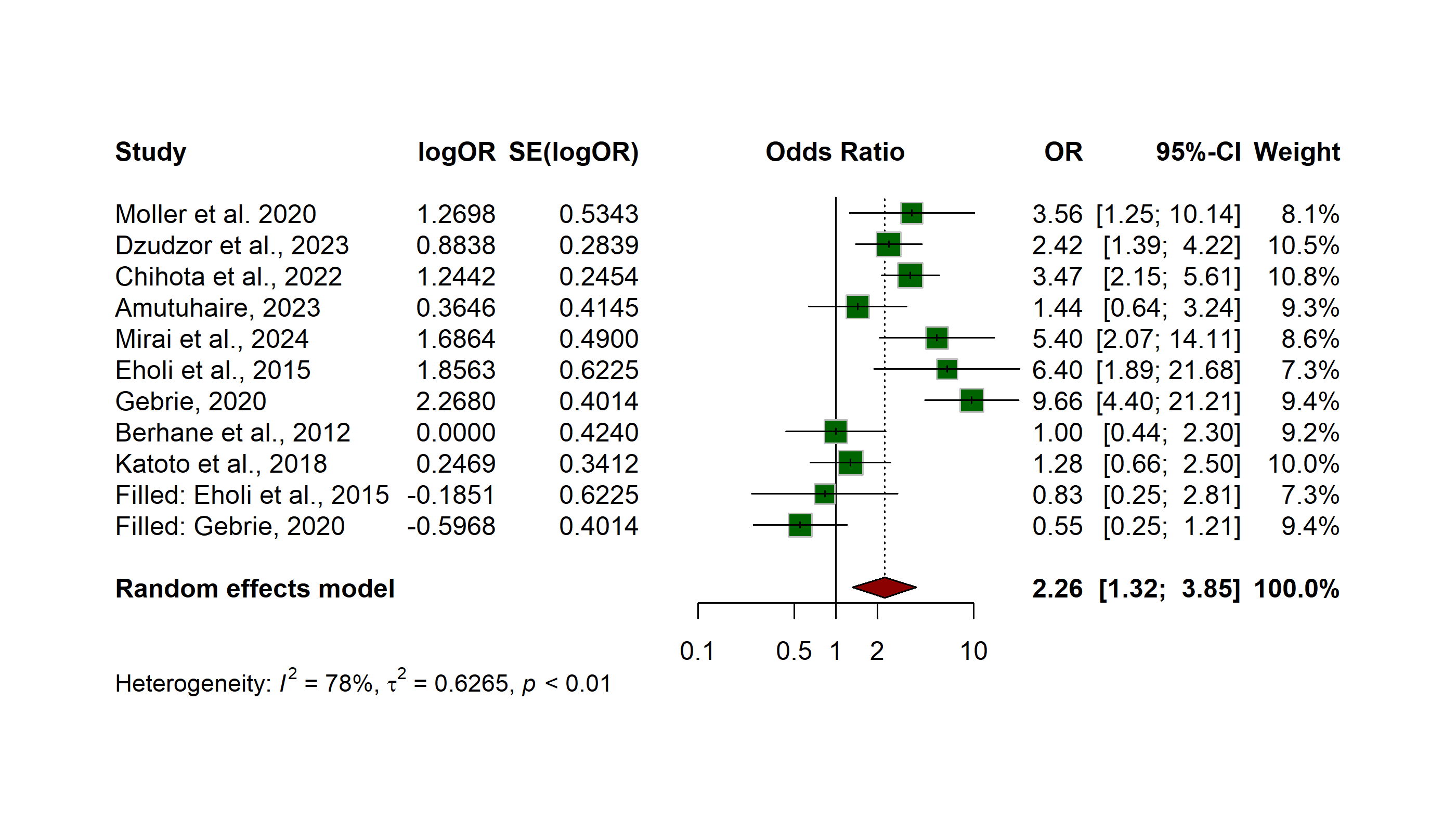


Figure S2a: Showing a Forest plot (Female sex) with Trim-and-Fill method which estimates the number of potentially missing studies and recalculates the pooled effect size


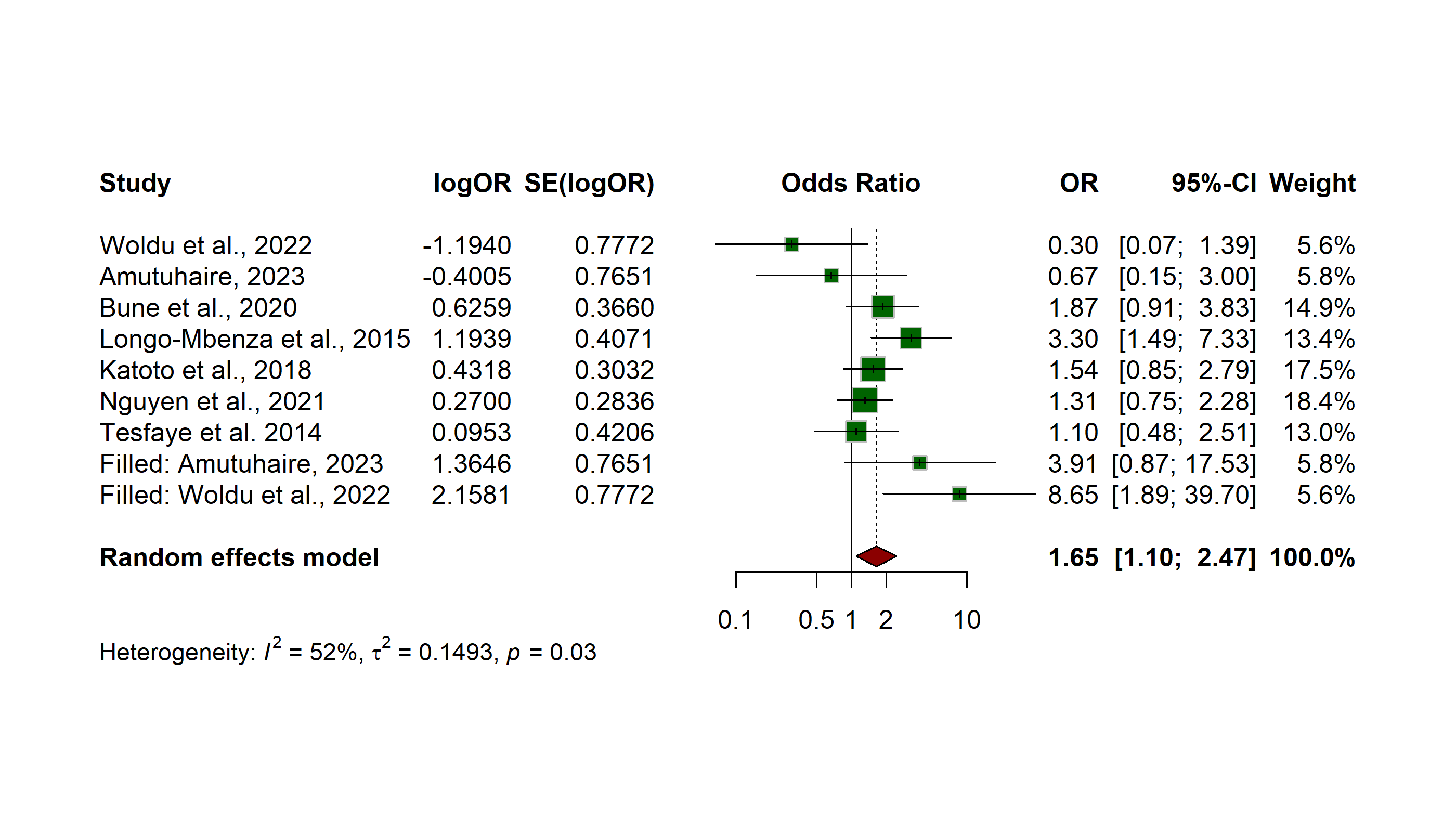


Figure S2b: Showing a Forest plot (Alcohol) with Trim-and-Fill method which estimates the number of potentially missing studies and recalculates the pooled effect size


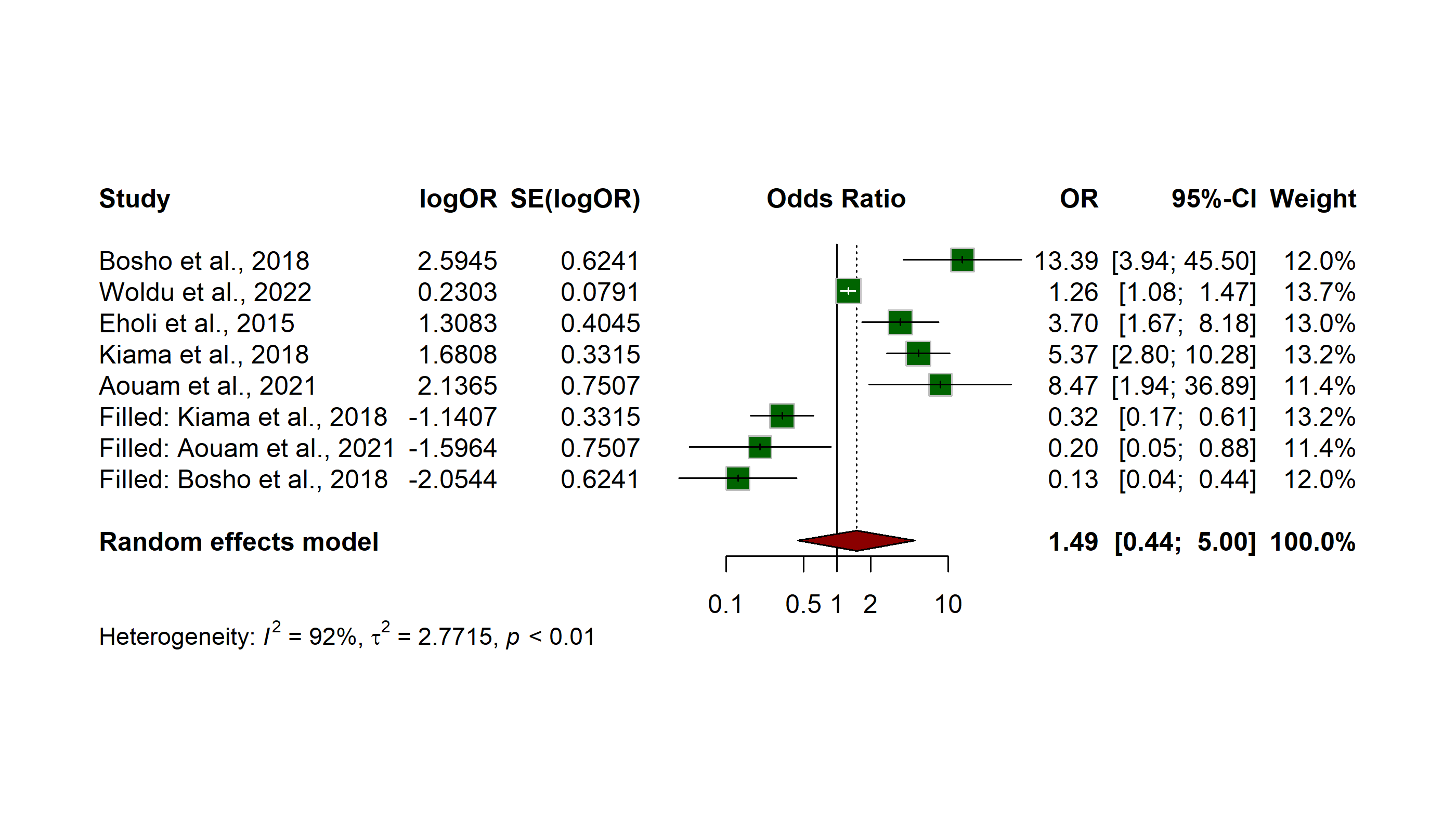


Figure S2c: Showing a Forest plot (BMI>25kg/m^2^) with Trim-and-Fill method which estimates the number of potentially missing studies and recalculates the pooled effect size


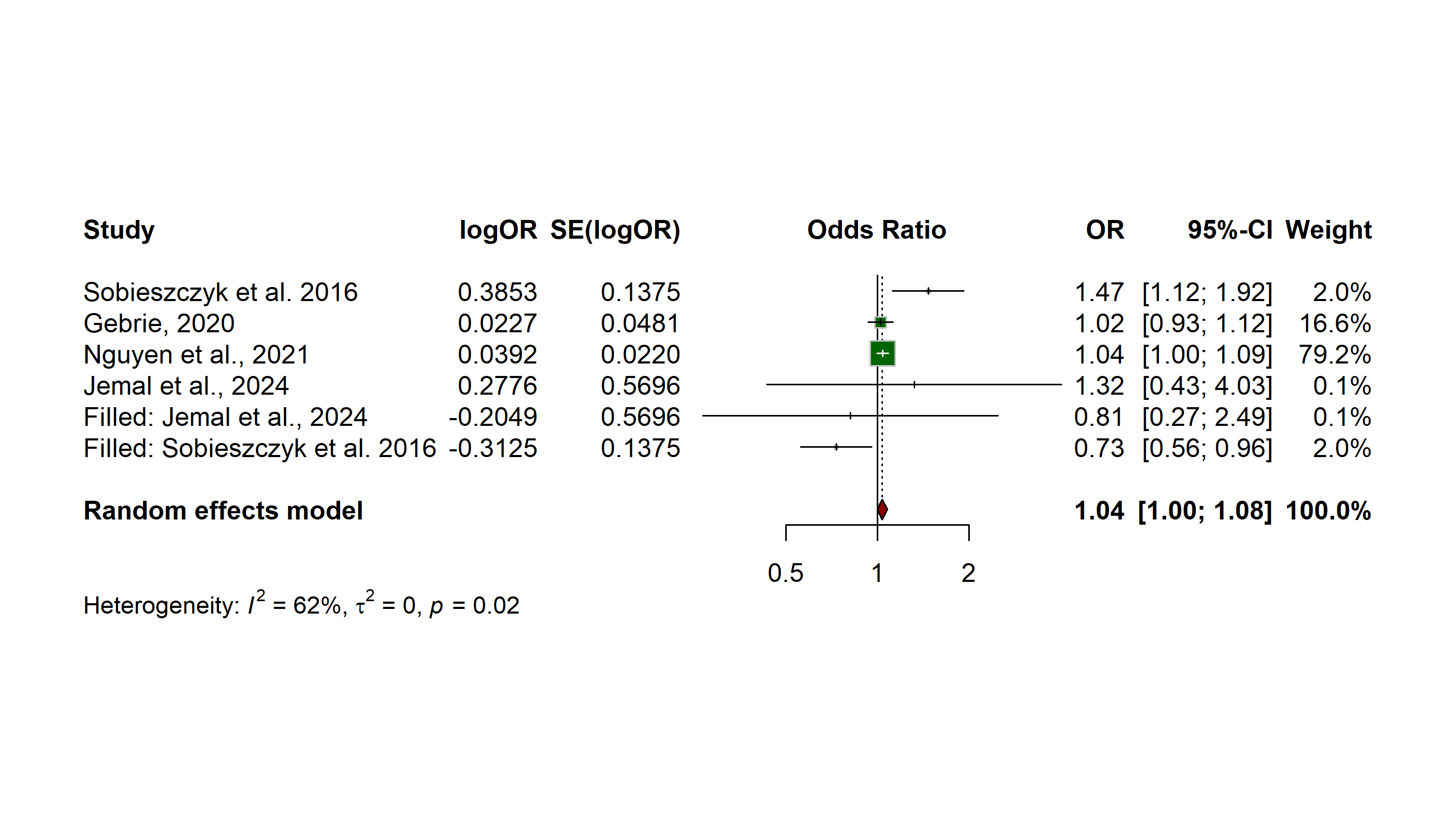


Figure S2d: Showing a Forest plot (HIV) with Trim-and-Fill method which estimates the number of potentially missing studies and recalculates the pooled effect size


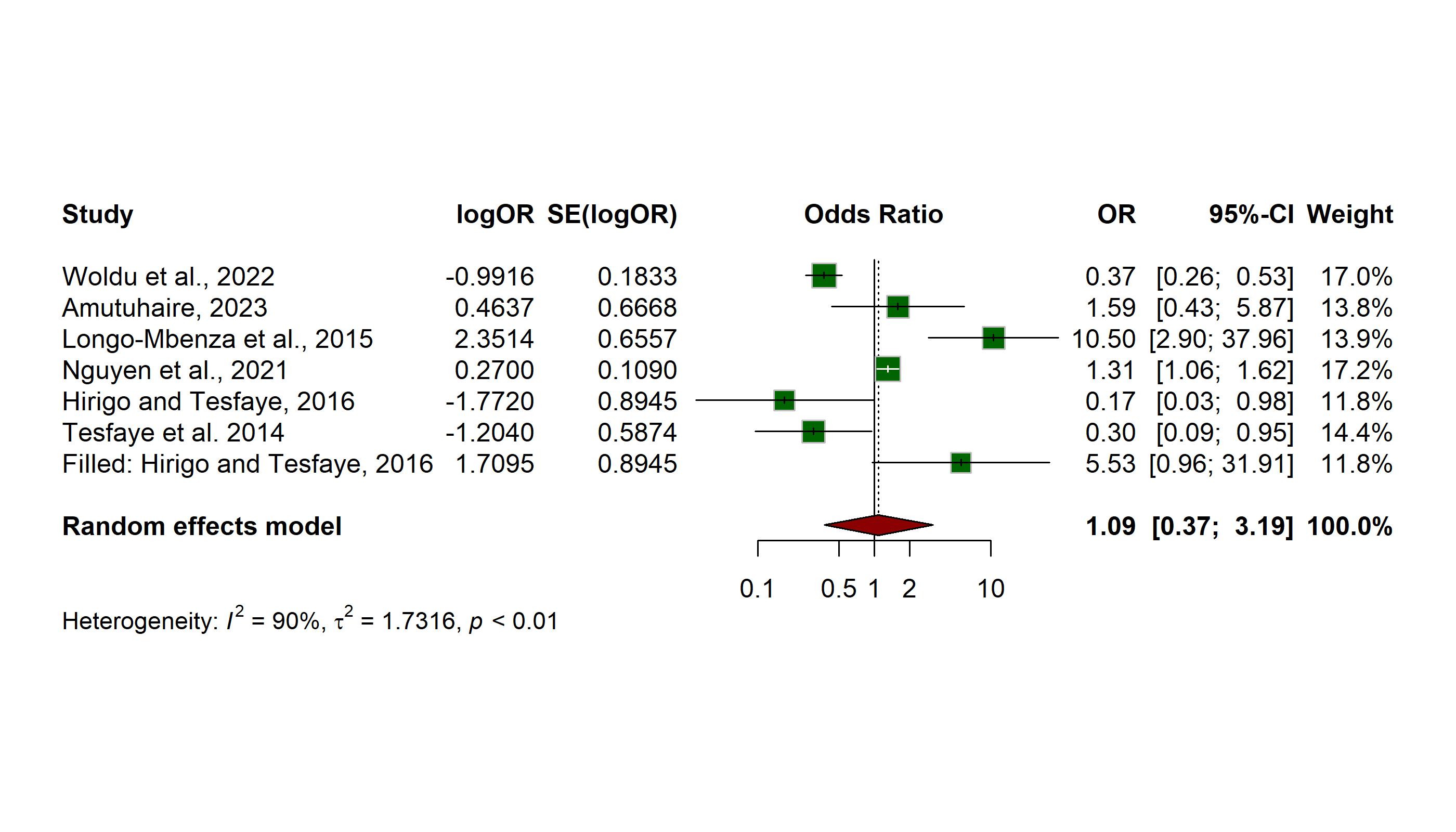


Figure S2e: Showing a Forest plot (Smoking) with Trim-and-Fill method which estimates the number of potentially missing studies and recalculates the pooled effect size


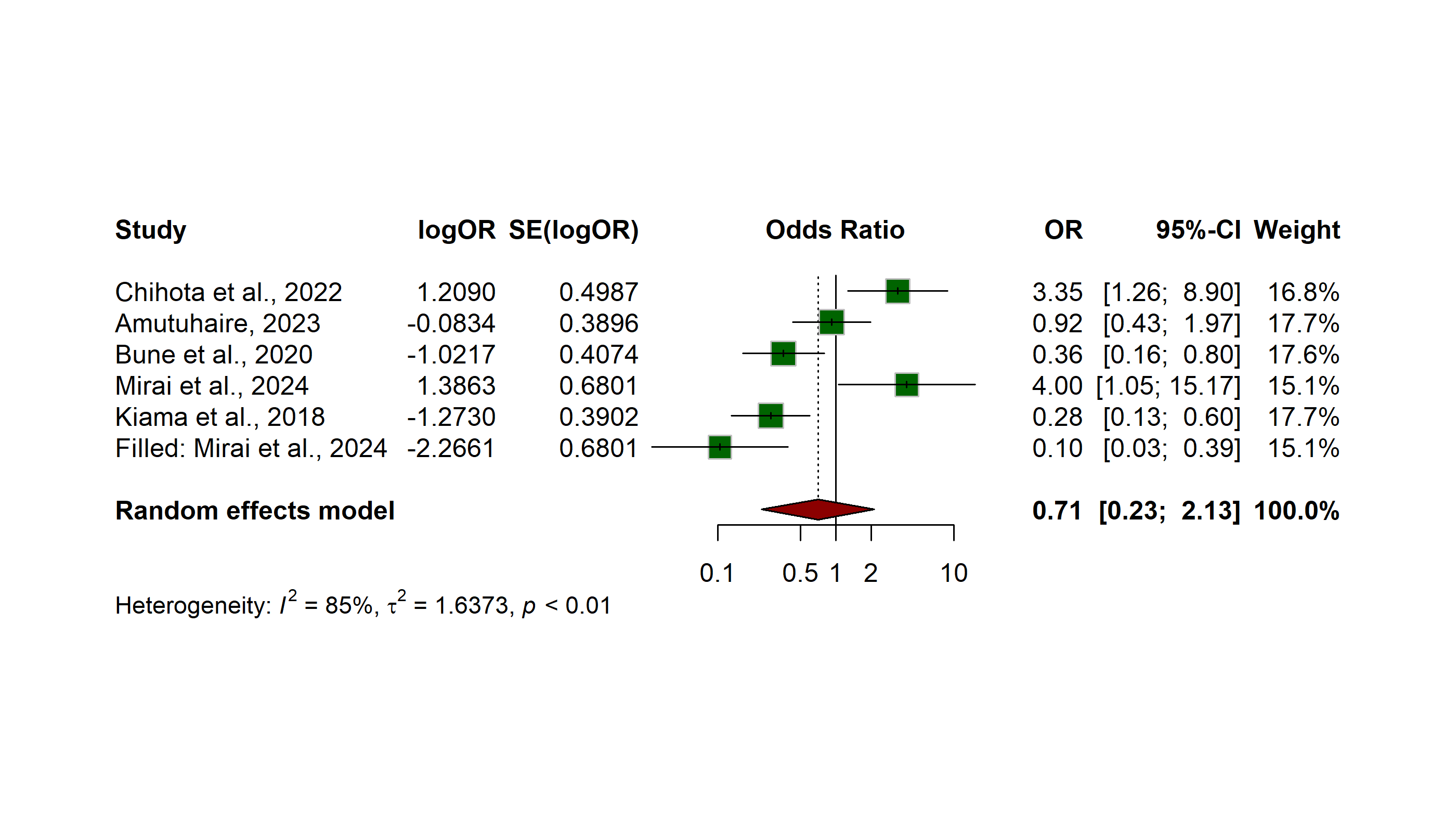


Figure S2f: Showing a Forest plot (Physical activity) with Trim-and-Fill method which estimates the number of potentially missing studies and recalculates the pooled effect size


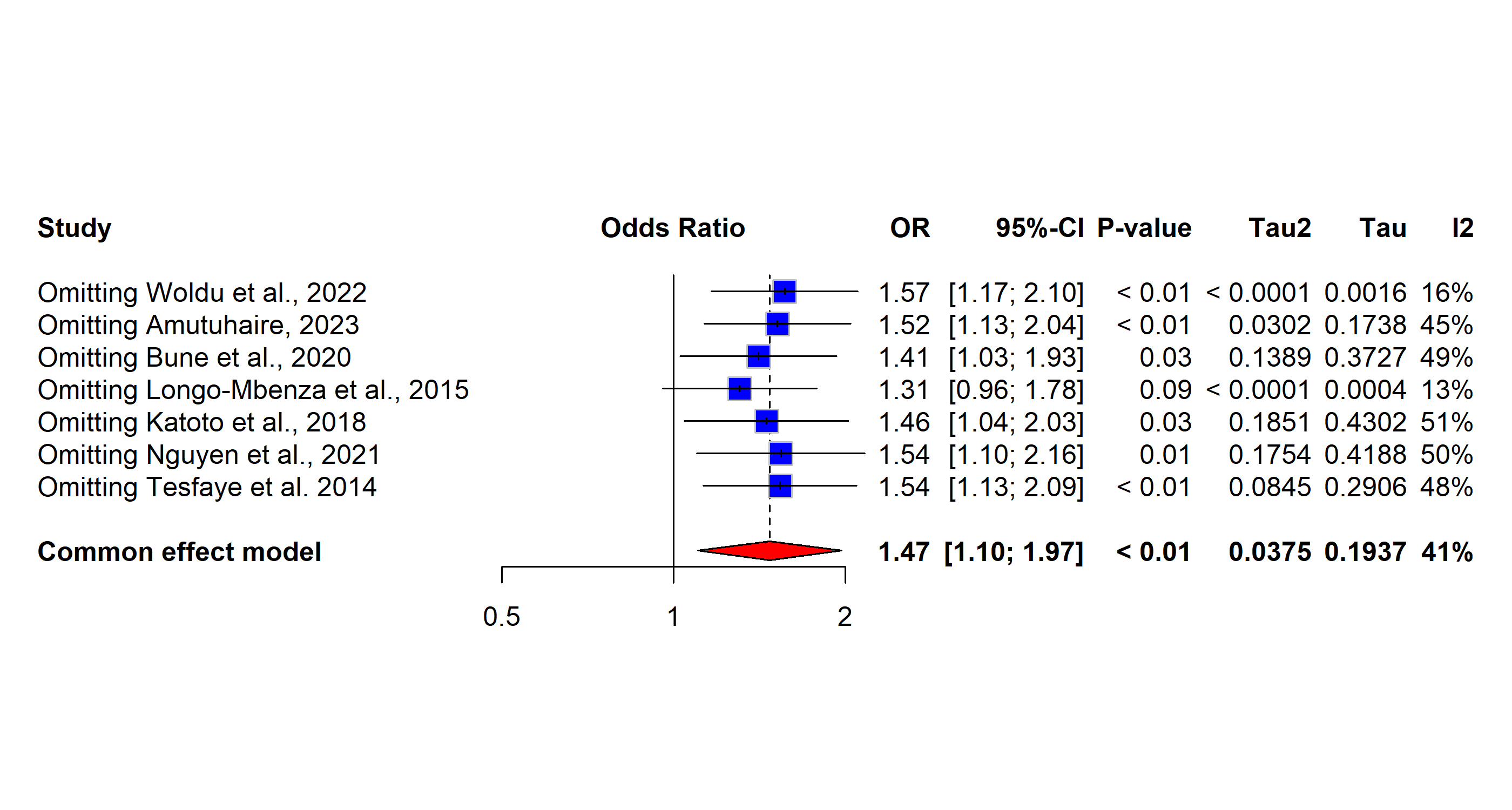


Figure S3a: Leave out sensitivity analyses for alcohol as a determinant to evaluate the robustness of study findings


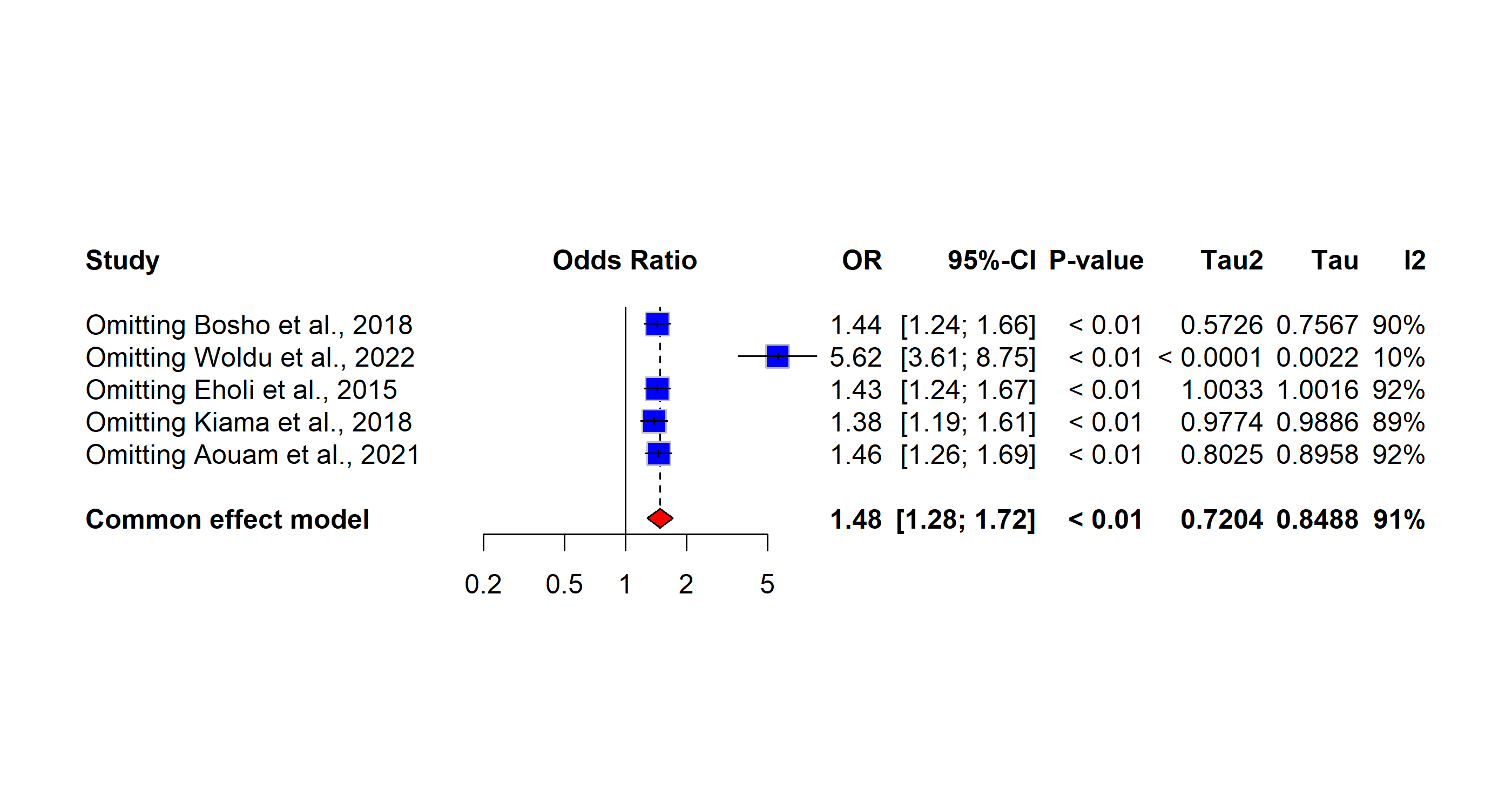


Figure S3b: Leave out sensitivity analyses for BMI as a determinant to evaluate the robustness of study findings


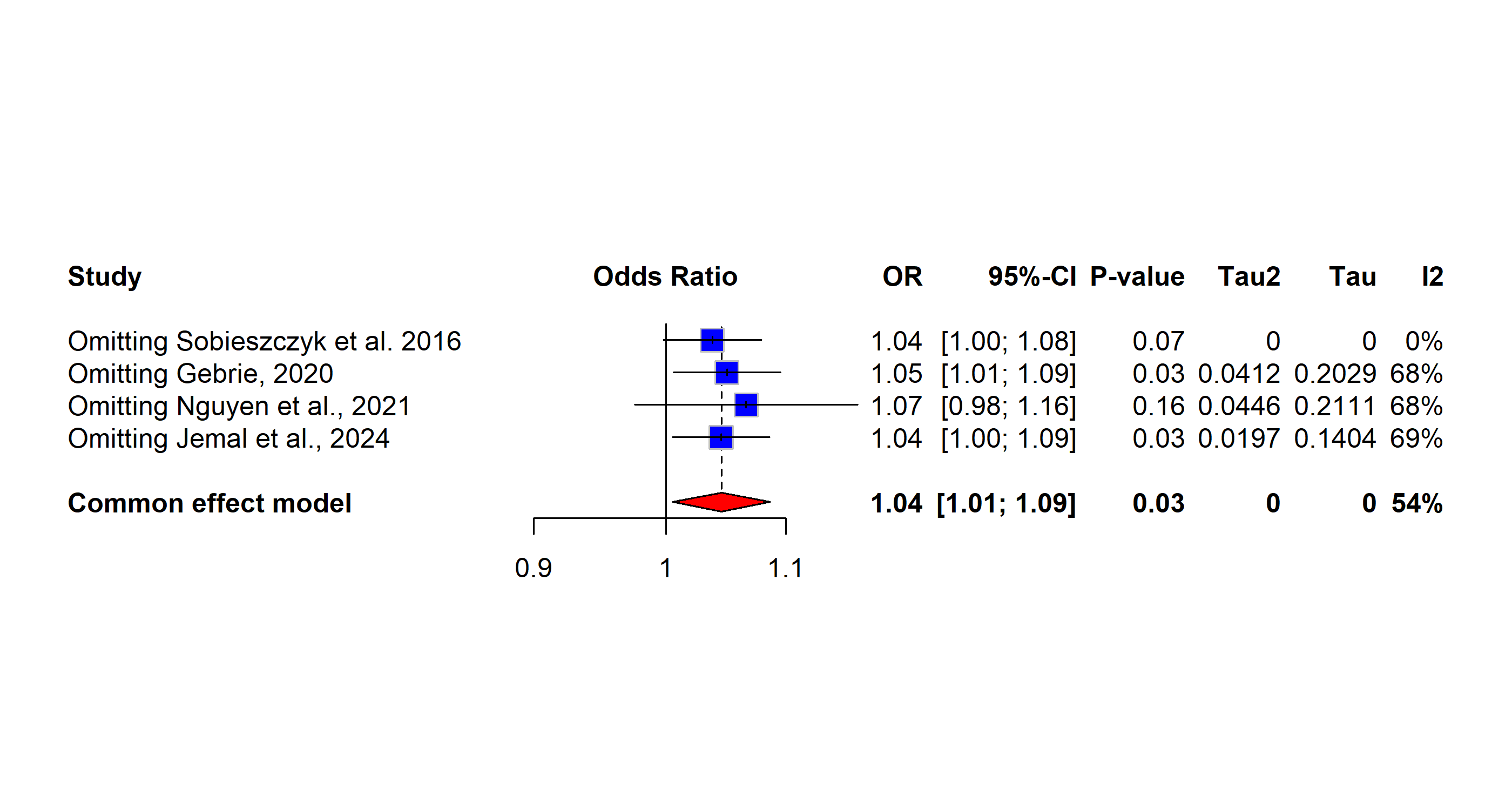


Figure S3c: Leave out sensitivity analyses for HIV as a determinant to evaluate the robustness of study findings


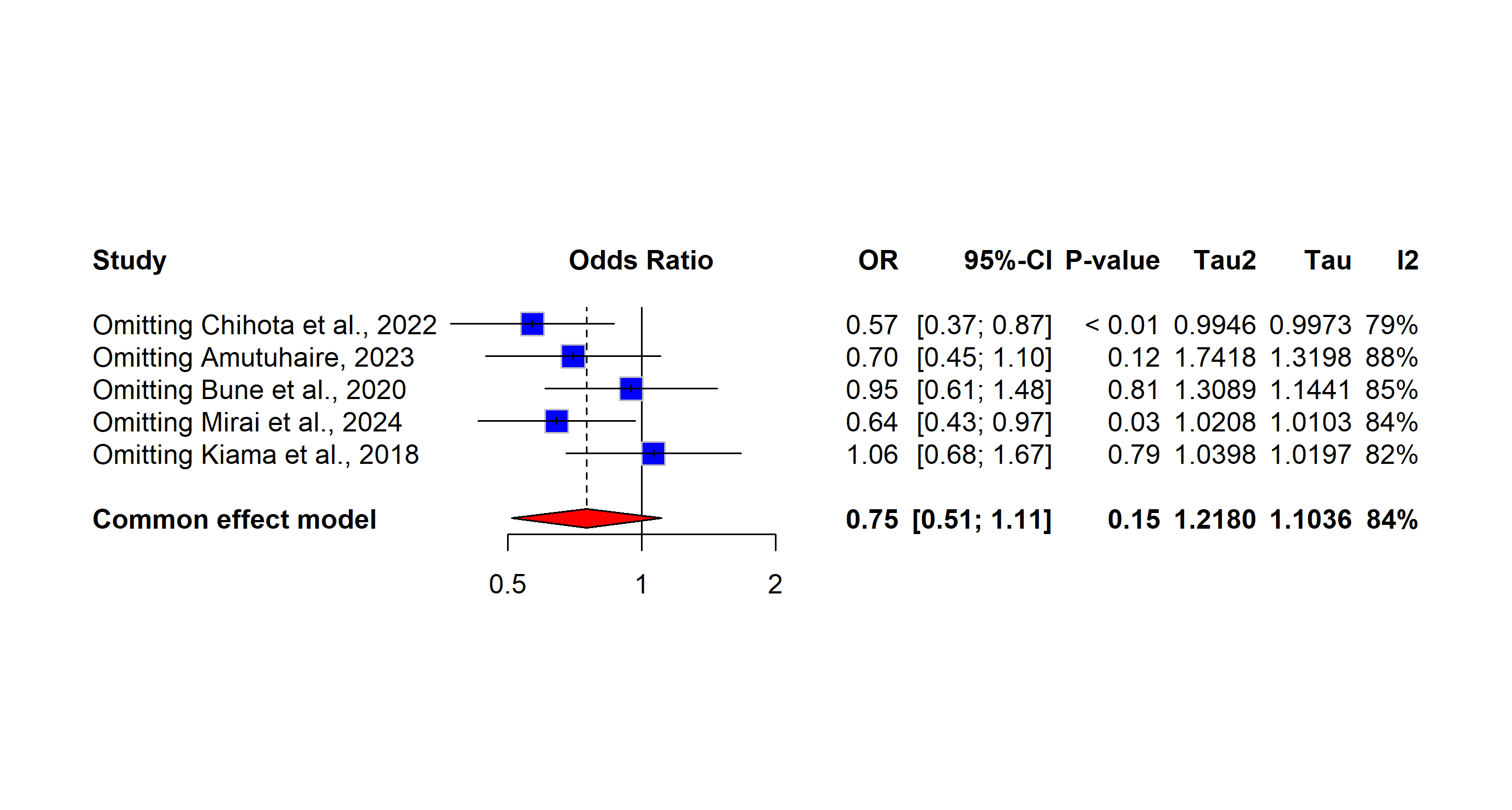


Figure S3d: Leave out sensitivity analyses for physical activity as a determinant to evaluate the robustness of study findings


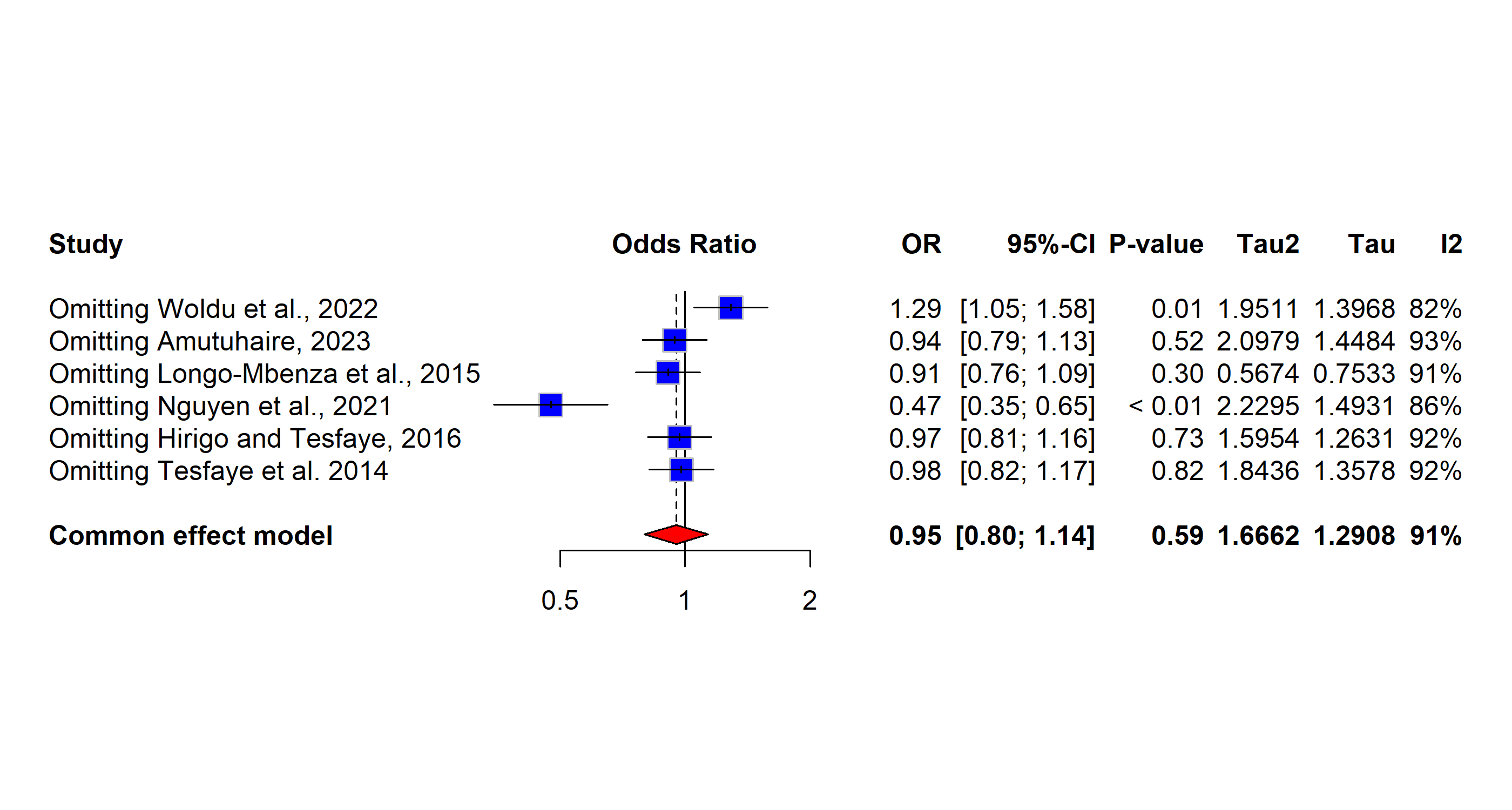


Figure S3e: Leave out sensitivity analyses for smoking as a determinant to evaluate the robustness of study findings


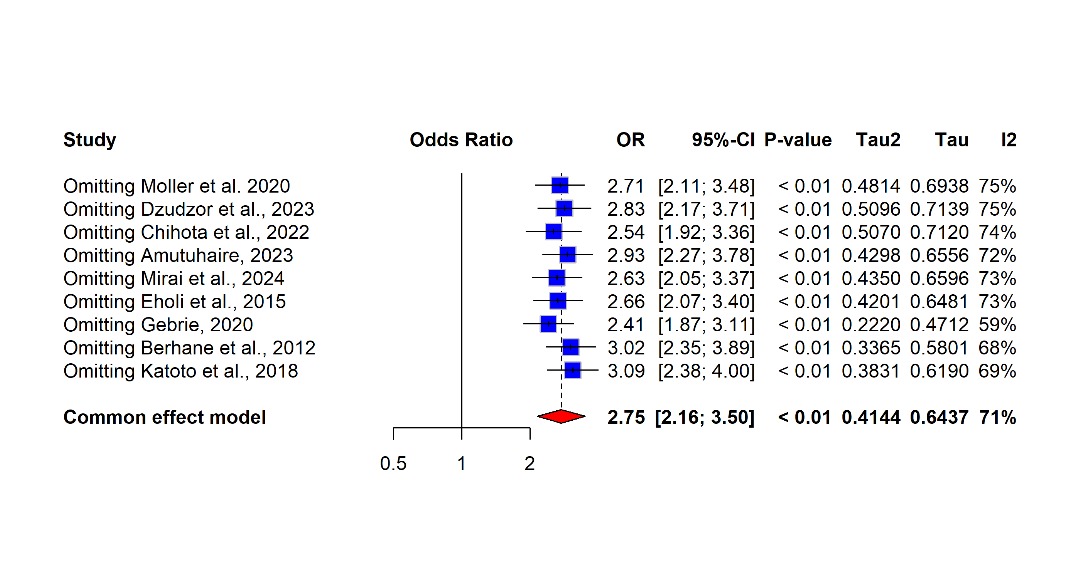


Figure S3f: Leave out sensitivity analyses for female sex as a determinant to evaluate the robustness of study findings
